# Bispecific antibodies promote natural killer cell-mediated elimination of HIV-1 reservoir cells

**DOI:** 10.1038/s41590-023-01741-5

**Published:** 2024-01-26

**Authors:** Nathan L. Board, Zhe Yuan, Fengting Wu, Milica Moskovljevic, Meghana Ravi, Srona Sengupta, Sung Soo Mun, Francesco R. Simonetti, Jun Lai, Pablo Tebas, Kenneth Lynn, Rebecca Hoh, Steven G. Deeks, Janet D. Siliciano, Luis J. Montaner, Robert F. Siliciano

**Affiliations:** 1 Department of Medicine, The Johns Hopkins University School of Medicine, Baltimore, MD USA; 2 The Wistar Institute, Philadelphia, PA USA; 3 Presbyterian Hospital-University of Pennsylvania Hospital, Philadelphia, PA USA; 4 Department of Medicine, University of California San Francisco, San Francisco, CA USA; 5 Howard Hughes Medical Institute, Baltimore, MD USA

**Keywords:** HIV infections, Immunotherapy, NK cells, HIV infections

## Abstract

The persistence of CD4^+^ T cells carrying latent human immunodeficiency virus-1 (HIV-1) proviruses is the main barrier to a cure. New therapeutics to enhance HIV-1-specific immune responses and clear infected cells will probably be necessary to achieve reduction of the latent reservoir. In the present study, we report two single-chain diabodies (scDbs) that target the HIV-1 envelope protein (Env) and the human type III Fcγ receptor (CD16). We show that the scDbs promoted robust and HIV-1-specific natural killer (NK) cell activation and NK cell-mediated lysis of infected cells. Cocultures of CD4^+^ T cells from people with HIV-1 on antiretroviral therapy (ART) with autologous NK cells and the scDbs resulted in marked elimination of reservoir cells that was dependent on latency reversal. Treatment of human interleukin-15 transgenic NSG mice with one of the scDbs after ART initiation enhanced NK cell activity and reduced reservoir size. Thus, HIV-1-specific scDbs merit further evaluation as potential therapeutics for clearance of the latent reservoir.

## Abstract (web summary)

Siliciano and colleagues describe the generation of bispecific antibodies that target the HIV-1 envelope protein (Env) on the surface of HIV-1-infected cells and the receptor CD16 on the surface of NK cells to induce the NK cell-mediated lysis of HIV-1-infected cells and reduce the viral reservoir.

## Main

ART suppresses replication of HIV-1 (refs. ^1,2^) but is not curative owing to the persistence of long-lived, resting memory CD4^+^ T cells with latent HIV-1 proviruses^3,4^. One approach to cure, known as ‘shock and kill’, involves selective reactivation of HIV-1 gene expression with latency reversing agents (LRAs) followed by immune-mediated clearance of infected cells^5,6^. To date, LRA-based clinical interventions have not achieved substantial reduction in the latent reservoir despite observed increases in plasma and cell-associated viral RNA^7–9^. A potential explanation is compromised function of cytolytic immune effector cells and inefficient elimination of cells expressing viral gene products^10–12^.

One approach to improving cell-mediated elimination of HIV-1-infected cells involves bispecific antibodies. Bispecific antibodies can promote CD8^+^ T cell- or NK cell-mediated killing of tumor cells and have been used successfully for treatment of various cancers^13^. Thus far, bispecific antibody-based immunotherapies targeting HIV-1-infected cells have focused on engaging CD8^+^ T cells^14–16^. However, sustained exposure to HIV-1 antigens may promote CD8^+^ T cell exhaustion^10,11^ and several LRAs suppress the cytolytic potential of CD8^+^ T cells^17–19^. In addition, cytokine release syndrome has been reported in several clinical trials of T cell-engaging bispecific antibodies^20,21^, giving rise to some safety concerns.

In the present study, we propose the use of NK cells as effector cells in an alternative bispecific antibody approach. NK cells mediate robust antibody-dependent cellular cytotoxicity (ADCC) against HIV-1-infected cells, and elimination of HIV-1-infected cells through ADCC has been reported in protective vaccine responses and in suppression of viral replication in elite controllers^6^. Furthermore, NK cell-based approaches have not been reported to induce cytokine release syndrome. In the present study, we show that two bispecific antibodies targeting the HIV-1 Env and the human type III Fcγ receptor (CD16) enhance HIV-1 specific NK cell activation and cytolysis in vitro and NK cell-mediated reservoir clearance ex vivo. In addition, we found that one of the bispecific antibodies promotes elimination of HIV-1 infected cells in a mouse model of HIV-1 infection.

## Results

### ScDbs induce robust NK cell activation

**Fig. 1:**
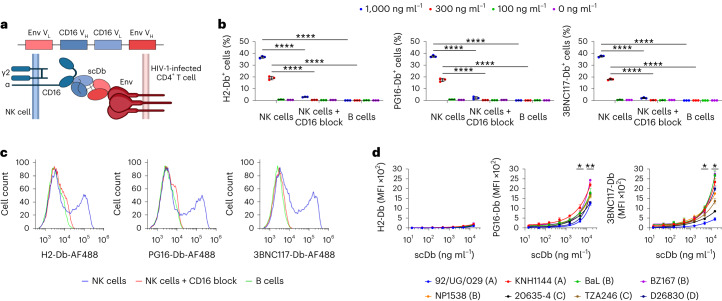
PG16-Db and 3BNC117-Db bind CD16 and Env. **a**, Schematic showing that the scDb consists of heavy- and light-chain variable regions of an Env-specific antibody and a CD16-specific antibody separated by glycine repeat linkers. ScDbs bind to CD16 expressed on the surface of cytolytic NK cells and to Env expressed on the surface of HIV-1-infected CD4^+^ T cells. **b**, Binding of H2-Db, PG16-Db and 3BNC117-Db to NK cells with or without blocking with a CD16-specific antibody (3G8) and to CD16^−^ B cells. Data are reported as the percentage of cells that stained positive for the scDb (mean, s.d.) from three replicates. The significance was determined by two-way ANOVA followed by Dunnett’s test for multiple comparisons, ^****^P < 0.0001. **c**, Representative flow histograms showing the binding of H2-Db, PG16-Db and 3BNC117-Db to NK and B cells as in **b** at concentrations of 1,000 ng ml^−1^. Data are reported as fluorescence intensity. **d**, Binding of H2-Db, PG16-Db and 3BNC117-Db to HEK293T cells transfected with *env* from th*e* HIV-1 isolates: 92/UG/092 (clade A), KNH1144 (clade A), BaL (clade B), BZ167 (clade B), NP1538 (clade B), 20635-4 (clade C), TZA246 (clade C) and D28630 (clade D). Data are reported as median fluorescence intensity (MFI) (mean, s.d.) from three replicates. The significance was determined by two-sided Student’s *t*-tests against a theoretical mean of 0% reduction: ^*^P < 0.05, ^**^P < 0.01 for each tested *env* isolate at the indicated concentration.

**Extended Data Fig. 1:**
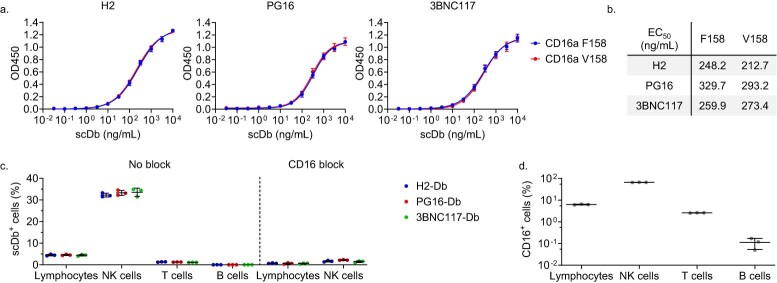
H2-Db, PG16-Db, and 3BNC117-Db bind both CD16a recombinant protein and cell surface CD16a. **a**, Binding of H2-Db, PG16-Db, 3BNC117-Db to immobilized CD16a recombinant proteins. Both CD16 variants are identical except for a single amino acid substitution at position 158 (F/V). Data reported as the optical density at 450nm (OD450) (mean, SD) from three replicates and were fitted to a dose-response nonlinear regression model. **b**, Calculated EC_50_ values for the fitted nonlinear regression model from **a** reported in ng/mL. **c**, Binding of H2-Db, PG16-Db, and 3BNC117-Db to peripheral blood lymphocytes, CD3^−^CD56^+^ NK cells, CD3^+^ T cells, and CD19^+^ B cells with or without blocking with a CD16-specific antibody (3G8). PBMCs were stained with 1000 ng/mL of H2-Db, PG16-Db, or 3BNC117-Db. Data reported as the percentage of cells that stained positive for the indicated scDb (mean, SD) from three replicates. **d**, Expression of CD16 on peripheral blood lymphocytes, CD3^-^CD56^+^ NK cells, CD3^+^ T cells, and CD19^+^ B cells. Data reported as the percentage of CD16^+^ cells (mean, SD) from three replicates.

We incorporated sequences of antibodies against Env and CD16 into scDbs. ScDbs consist of a single polypeptide chain encoding the heavy- and light-chain variable regions of two antibodies separated by flexible glycine linkers, which assemble to form two distinct, functional, single-chain variable fragment (scFv) domains^16,22^ (Fig. 1a). Two HIV-1-specific scDbs were designed based on the broadly neutralizing antibodies (bNAbs) PG16 (ref. ^23^) and 3BNC117 (ref. ^24^), which are specific for the apex region and the CD4-binding site of Env, respectively (Supplementary Table 1 and Supplementary Fig. 1). Both bNAbs were reported to recognize diverse HIV-1 Env isolates and eliminate HIV-1-infected cells through ADCC^25,26^. PG16 and 3BNC117 were coupled with the CD16 antibody NM3E2 to generate PG16-Db and 3BNC117-Db (Supplementary Table 1 and Supplementary Fig. 1). We also generated an irrelevant isotype control scDb called H2-Db, which incorporates an antibody directed against a mutant p53 epitope presented on HLA-A*02:01 (ref. ^22^) combined with NM3E2. All three scDbs bound CD16 recombinant proteins (Extended Data Fig. 1a,b) and cell-surface CD16 expressed on primary human NK cells (Fig. 1b,c and Extended Data Fig. 1c,d). H2-Db, PG16-Db and 3BNC117-Db did not bind to NK cells preincubated with a CD16-specific antibody or to B cells that lack CD16 expression (Fig. 1b,c and Extended Data Fig. 1c,d). In addition, PG16-Db and 3BNC117-Db bound Env presented on the surface of HEK293T cells transfected with *env* genes from a diverse set of HIV-1 isolates (Fig. 1d and Supplementary Fig. 2), indicating that both HIV-1-specific scDbs retained the multiple HIV-1 subtype breadth of Env binding exhibited by their parental bNAbs. H2-Db did not bind transfected HEK293T cells. Thus, PG16-Db and 3BNC117-Db specifically bound HIV-1 Env and CD16 in the expected manner.

**Fig. 2:**
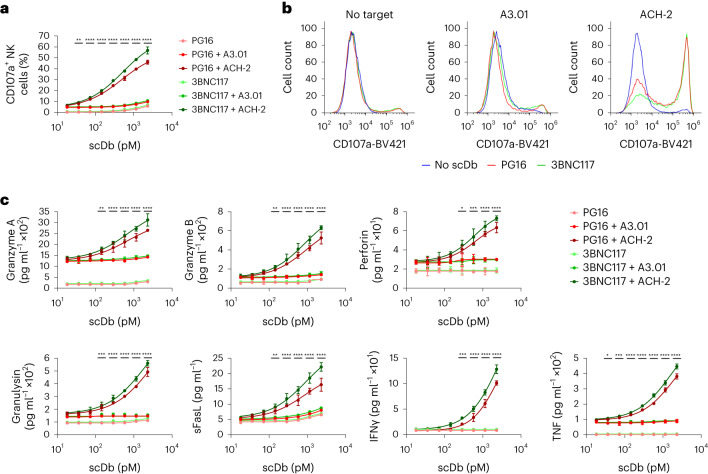
PG16-Db and 3BNC117-Db induce polyfunctional HIV-1-specific NK cell responses. **a**, Expression of CD107a on CD3^−^CD56^+^ NK cells cultured alone or with HIV-1-uninfected A3.01 cells or HIV-1-infected ACH-2 cells in the presence of H2-Db, PG16-Db and 3BNC117-Db. Data are reported as the percentage of cells that stained positive for CD107a (mean, s.d.) from three replicates. The significance was determined by two-way ANOVA followed by Dunnett’s test for multiple comparisons: ^**^P < 0.01, ^****^P < 0.0001 comparing the A3.01 with the ACH-2 conditions for both scDbs at the indicated concentrations. **b**, Representative flow histograms for CD107a expression on CD3^−^CD56^+^ NK cells as in **a**. Data are reported as fluorescence intensity. **c**, Expression of granzyme A, granzyme B, perforin, granulysin, sFasL, IFNγ and TNF effector molecules in the supernatants of NK cells cultured alone or with HIV-1-uninfected A3.01 cells or HIV-1-infected ACH-2 cells as in **a**. Data are reported as concentration (mean, s.d.) from three replicates. The significance was determined by two-way ANOVA followed by Dunnett’s test for multiple comparisons: ^*^P < 0.05, ^**^P < 0.01, ^***^P < 0.001, ^****^P < 0.0001 comparing the A3.01 with the ACH-2 conditions for both PG16 and 3BNC117 scDbs at the indicated concentration.

We next assessed the ability of the scDbs to induce specific activation of NK cells. NK cells from HIV-1-negative participants were cocultured for 6 h with tumor necrosis factor (TNF)-pretreated ACH-2 cells in the presence of PG16-Db or 3BNC117-Db. ACH-2 cells are transformed CD4^+^ T cells which carry an HIV-1 provirus that can be induced with TNF and have been used as a model of HIV-1 latency^27^. Addition of PG16-Db or 3BNC117-Db resulted in dose-dependent increases in NK cell expression of CD107a, a marker of degranulation (Fig. 2a,b). Neither PG16-Db nor 3BNC117-Db induced NK cell expression of CD107a in cocultures with the TNF-pretreated, uninfected. parental T cell line A3.01 (Fig. 2a,b), indicating improved cytolytic degranulation in response to the HIV-1-infected target cells. NK cell expression of CD107a was greatly enhanced by PG16-Db and 3BNC117-Db compared with equimolar concentrations of the respective parental IgG molecules, termed here PG16-Ig and 3BNC117-Ig, in NK cell cocultures with TNF-pretreated ACH-2 cells (Supplementary Fig. 3). PG16-Db and 3BNC117-Db induced only minimal NK cell degranulation in the absence of target cells (Fig. 2a).

In addition, we assessed indicators of NK cell activation including cytolytic granule proteins (granzyme A, granzyme B, perforin and granulysin), a soluble TNF family death ligand (sFasL) and inflammatory cytokines (interferon (IFN)-γ, TNF) in the supernatants of the same cultures. At concentrations between 10 and 1,000 pM, PG16-Db and 3BNC117-Db elicited dose-dependent increases in supernatant concentrations of these effector molecules in the presence of HIV-1-infected (ACH-2), but not uninfected (A3.01), target cells (Fig. 2c). Even at concentrations <300 pM, the scDbs induced higher release of all assayed effector molecules when cultured with ACH-2 cells than in the corresponding A3.01 cocultures (Fig. 2c). Notably, PG16-Db and 3BNC117-Db did not increase the production of effector molecules in the absence of Env-expressing ACH-2 cells (Fig. 2c). Together, these results indicated that PG16-Db and 3BNC117-Db potently induced polyfunctional NK cell activity in the presence of Env-expressing target cells.

### ScDbs induce NK cell-mediated elimination of HIV-1^+^ cells

**Fig. 3:**
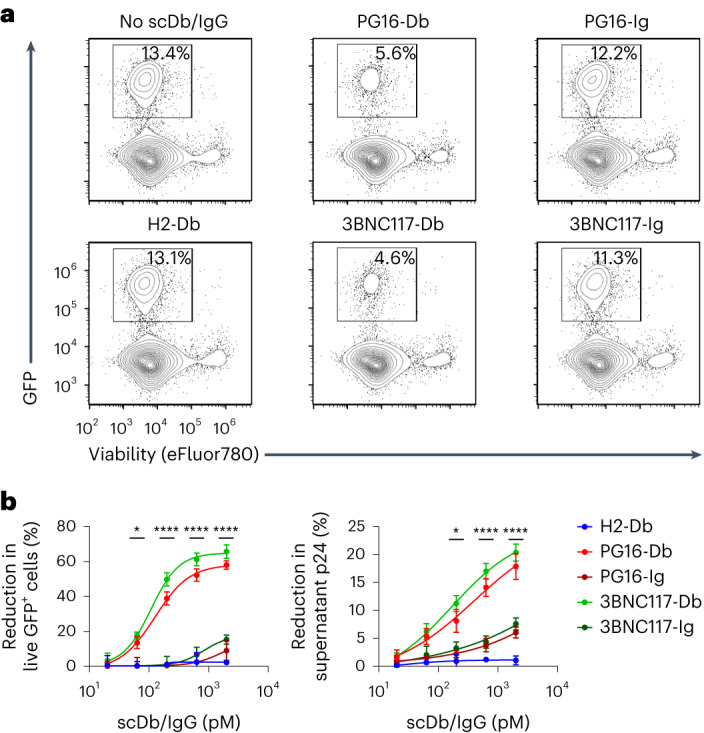
PG16-Db and 3BNC117-Db promote NK cell-mediated elimination of HIV-1-infected cells. **a**, Representative flow plots showing the percentage of live GFP^+^CD4^+^ T cells after coculture with autologous NK cells alone or in the presence of 2,000 pM of H2-Db, PG16-Db, 3BNC117-Db, PG16-Ig or 3BNC117-Ig. **b**, Frequency of live GFP^+^CD4^+^ T cells (left) or p24 supernatant concentration (right) after coculture with autologous NK cells as in **a** relative to no scDb/IgG treatment. Data are reported as a percentage reduction (mean, s.d.) from three replicates. The significance was determined by two-way ANOVA followed by Dunnett’s test for multiple comparisons: ^*^P < 0.05, ^****^P < 0.0001 comparing the scDb with the IgG conditions for both PG16 and 3BNC117 at the indicated concentrations.

**Extended Data Fig. 2:**
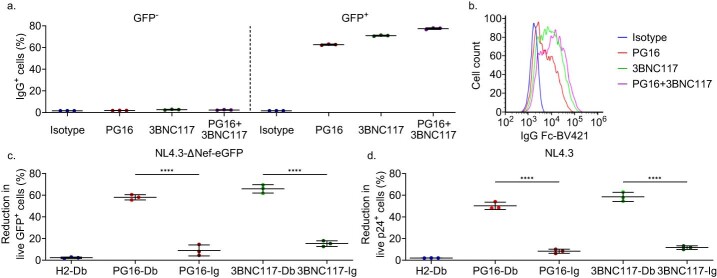
PG16-Db and 3BNC117-Db promote elimination of Env^+^ cells. **a**, Binding of PG16-Ig and 3BNC117-Ig to NL4.3-ΔNef-eGFP-infected CD4^+^ T cells. Data reported as the percentage of hIgG Fc^+^ CD4^+^ T cells after preincubation of the cells with 20 μg/mL of PG16-Ig, 3BNC117-Ig, or an equimolar combination of PG16-Ig+3BNC117-Ig (mean, SD) from three replicates. **b**, Representative flow histograms showing binding of PG16-Ig, 3BNC117-Ig, and PG16-Ig+3BNC117-Ig to GFP^+^ CD4^+^ T cells. All conditions shown are after pre-incubation of the infected CD4^+^ T cells with 20 μg/mL of the corresponding IgG. Data reported as fluorescence intensity. **c**, CD4^+^ T cells infected with NL4.3- ΔNef-eGFP were co-cultured with autologous NK cells in the presence of H2-Db, PG16-Db, 3BNC117-Db, PG16-Ig, or 3BNC117-Ig at 2000 pM. Data reported as the frequency of viable GFP^+^ cells relative to no scDb/IgG treatment (percent reduction, mean, SD) from three replicates. Significance determined by one-way ANOVA followed by Dunnett’s test for multiple comparisons, ****P < 0.0001. **d**, CD4^+^ T cells infected with NL4.3 were co-cultured with autologous NK cells in the presence of H2-Db, PG16-Db, 3BNC117-Db, PG16-Ig, or 3BNC117-Ig. Data reported as the frequency of viable p24^+^ cells relative to no scDb/IgG treatment (percent reduction, mean, SD) from three replicates. Significance determined by one-way ANOVA followed by Dunnett’s test for multiple comparisons, ****P < 0.0001.

To test the ability of the scDbs to promote killing of HIV-1-infected cells, CD4^+^ T cells from HIV^−^ participants were activated with CD3 + CD28 antibodies and infected with a replication-competent HIV-1 construct carrying enhanced green fluorescent protein (eGFP) in the open reading frame of the *nef* gene (NL4.3^−^ΔNef-eGFP). PG16-Ig and 3BNC117-Ig bound viable GFP^+^CD4^+^ T cells (Extended Data Fig. 2a,b), indicating that the infected CD4^+^ T cells expressed Env. Infected CD4^+^ T cells were then cocultured with autologous NK cells at an effector:target (E:T) ratio of 1:3 in the presence of the scDbs or their parental immunoglobulin G (IgG) molecules. After 18 h, we observed strong, dose-dependent decreases in viable GFP^+^CD4^+^ T cells with both PG16-Db and 3BNC117-Db compared with H2-Db or no scDb (Fig. 3a,b and Extended Data Fig. 2c). At 2,000 pM, PG16-Db and 3BNC117-Db mediated 58% and 66% reductions in the number of viable GFP^+^ cells compared with no scDb treatment, respectively (Fig. 3b). Equimolar concentrations of PG16-Ig and 3BNC117-Ig resulted in 9% and 15% reductions in GFP^+^CD4^+^ T cells, respectively (Fig. 3b). There was no significant reduction in viable GFP^+^CD4^+^ T cells with H2-Db at any concentration tested (Fig. 3b). In addition, coculture of NK cells and autologous CD4^+^ T cells infected with a replication-competent HIV-1 construct that has a functional *nef* gene (NL4.3) resulted in 50% and 58% reductions in viable CD4^+^ T cells expressing HIV-1 p24 antigen with PG16-Db and 3BNC117-Db, respectively (Extended Data Fig. 2d). At a concentration of 2,000 pM PG16-Db and 3BNC117-Db resulted in 18% and 20% decreases in p24 in the supernatant, respectively, compared with the no scDb control (Fig. 3b), consistent with the scDb-mediated elimination of HIV-1-infected cells. The reduction in supernatant p24 with H2-Db was negligible (1%), whereas those induced by PG16-Ig and 3BNC117-Ig were detectable (6% and 7%), but weaker than those induced by PG16-Db and 3BNC117-Db (Fig. 3b). The dose–response curves for viable GFP^+^CD4^+^ T cells and supernatant p24 indicated that PG16-Db and 3BNC117-Db were at least 1–2 log more potent than the corresponding IgG molecules in mediating the killing of HIV-1-infected CD4^+^ T cells (Fig. 3b), probably because the CD16-specific component of the scDbs had a much higher affinity for CD16 than the Fc domain of IgG molecules^28^. These results showed that PG16-Db and 3BNC117-Db promoted efficient NK cell-mediated killing of HIV-1-infected CD4^+^ T cells and did so at much lower concentrations than the parental IgG molecules.

### ScDbs induce ex vivo reduction of HIV-1 reservoirs

**Fig. 4:**
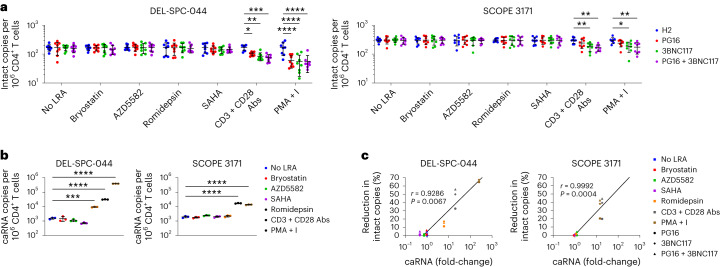
PG16-Db and 3BNC117-Db eliminate cells harboring intact proviruses when paired with strong latency reversal. **a**, Frequency of intact provirus^+^ CD4^+^ T cells after treatment with no LRA, bryostatin, AZD5582, romidepsin, SAHA, CD3 + CD28 antibodies or PMA + I and coculture with autologous NK cells in the presence of H2-Db, PG16-Db, 3BNC117-Db or PG16-Db + 3BNC117-Db. Data are reported as intact HIV-1 copies per million viable CD4^+^ T cells (mean, s.d.) from eight replicates. The significance was determined by two-way ANOVA followed by Dunnett’s test for multiple comparisons: ^*^P < 0.05, ^**^P < 0.01, ^***^P < 0.001, ^****^P < 0.0001. **b**, Expression of caRNA after treatment with LRAs as in **a**. Data are reported as caRNA copies per million CD4^+^ T cells (mean, s.d.) from three replicates. The significance was determined by one-way ANOVA followed by Dunnett’s test for multiple comparisons: ^***^P < 0.001, ^****^P < 0.0001. **c**, Correlation between percentage reduction in intact copies and mean fold-change in caRNA. Spearman’s *r* value is shown.

We next tested whether PG16-Db and 3BNC117-Db promoted the elimination of latently infected CD4^+^ T cells from people on long-term ART after latency reversal ex vivo. Peripheral blood mononuclear cells (PBMCs) were collected from 11 male participants (median age = 56 years, range = 34–69 years) with undetectable plasma HIV-1 RNA levels (<50 copies per ml) for >72 months (median time on ART = 289 months, range = 130–360 months; median time on suppressive ART = 185 months, range = 79–275 months; see Supplementary Table 2 for ART regimens). Primary CD4^+^ T cells from two participants were stimulated with a panel of LRAs for 18 h, followed by coculture with autologous NK cells at an E:T ratio of 1:3 in the presence of PG16-Db, 3BNC117-Db or an equimolar combination of both for 18 h. CD4^+^ T cells were re-isolated and depleted of apoptotic cells by immunomagnetic selection of annexin-V^+^ cells to prevent measurement of HIV-1 DNA from cells killed during coculture. The frequency of intact provirus^+^ cells was measured by the intact proviral DNA assay (IPDA), a droplet digital (dd)PCR assay that quantifies proviruses that lack major defects, such as large internal deletions and APOBEC3^−^-mediated hypermutation. No significant reduction in intact provirus^+^ cells was observed in CD4^+^ T cells incubated with the no LRA control, the protein kinase C agonist bryostatin, the SMAC mimetic AZD5582 or the histone deacetylase (HDAC) inhibitors romidepsin and SAHA in the presence of PG16-Db, 3BNC117-Db or PG16-Db + 3BNC117-Db compared with the H2-Db control (Fig. 4a). However, when CD4^+^ T cells were treated with agents that induce global T cell activation, such as CD3 + CD28 antibodies or phorbol myristate acetate + ionomycin (PMA + I), there were significant reductions of up to 56% and 67%, respectively, in the frequency of intact provirus^+^ CD4^+^ T cells were attained with PG16-Db + 3BNC117-Db relative to H2-Db (Fig. 4a).

To determine whether the reduction in intact provirus^+^ CD4^+^ T cells was dependent on the degree of latency reversal, we performed paired measurements of cell-associated polyadenylated HIV-1 RNA (caRNA) in aliquots of LRA-treated CD4^+^ T cells before NK cell coculture. Of the six LRAs tested, only CD3 + CD28 antibodies and PMA + I resulted in a significant increase in caRNA transcripts compared with the untreated control in both participants (Fig. 4b), consistent with prior reports^29^. The HDAC inhibitor romidepsin elicited a significant increase in caRNA transcripts in one of two participants (designated as DEL-SPC-044), but the corresponding reductions in intact provirus^+^ cells relative to the H2-Db control (11% with PG16-Db, 17% with 3BNC117-Db and 16% with PG16-Db + 3BNC117-Db) in this participant did not reach statistical significance (Fig. 4a). We found a significant correlation between caRNA induction and the reduction in intact provirus^+^ cells (Fig. 4c), suggesting that scDb-mediated elimination of intact provirus^+^ cells depended on efficient latency reversal.

**Fig. 5:**
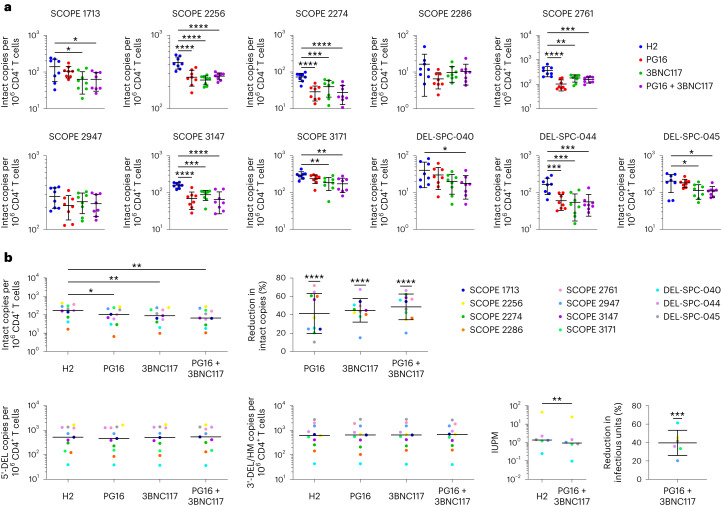
PG16-Db and 3BNC117-Db eliminate cells harboring intact and inducible proviruses. **a**, Frequency of intact provirus^+^CD4^+^ T cells after treatment with PMA + I and coculture with autologous NK cells in the presence of H2-Db, PG16-Db, 3BNC117-Db or PG16-Db + 3BNC117-Db. Data are reported as intact HIV-1 copies per million viable CD4^+^ T cells (mean, s.d.) from eight replicates. The significance was determined by one-way ANOVA followed by Dunnett’s test for multiple comparisons: ^*^P < 0.05, ^**^P < 0.01, ^***^P < 0.001, ^****^P < 0.0001. **b**, Average frequencies of intact provirus^+^ CD4^+^ T cells (top left), 5′-deleted provirus^+^ CD4^+^ T cells (bottom far left) and 3′-deleted/hypermutated provirus^+^CD4^+^ T cells (bottom middle left) after treatment with PMA + I and coculture with autologous NK cells as in **a** (mean, s.d.) across all 11 participants assessed. The significance was determined by one-way ANOVA followed by Dunnett’s test for multiple comparisons: ^*^P < 0.05, ^**^P < 0.01. Average IUPM viable CD4^+^ T cells (bottom middle right) after treatment with PMA + I and coculture with autologous NK cells as in **a** (mean, s.d.) across six participants assessed. The significance was determined by paired, two-sided Student’s *t*-test: ^**^P < 0.01. Average frequency of intact provirus^+^ CD4^+^ T cells (top middle) or average IUPM viable CD4^+^ T cells (bottom far right) relative to H2-Db. Data reported as a percentage reduction (mean, s.d.) from eleven or six participants, respectively. The significance was determined by Student’s *t*-tests against a theoretical mean of 0% reductions: ^***^P < 0.001, ^****^P < 0.0001.

**Extended Data Fig. 3:**
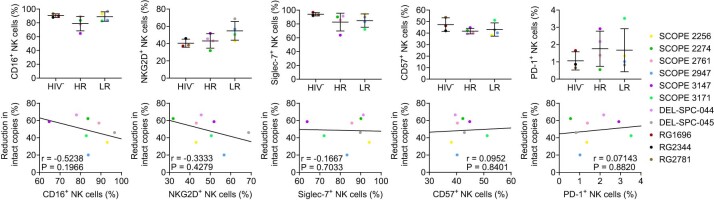
Efficacy of scDb-mediated reduction intact proviruses^+^CD4^+^ T cells cannot be attributed to NK cell phenotype. Expression of CD16, NKG2D, Siglec-7, CD57, and PD-1 on CD3^-^CD56^+^ NK cells. Participants were categorized into two subgroups, high response (HR, n = 4) and low response (LR, n = 4), based on whether that individual had an above or below average reduction in intact provirus^+^ cells with PG16-Db+3BNC117-Db. HIV^-^ participants were included for comparison (n = 3). Data reported as the percentage of cells that stained positive for the indicated marker (mean, SD). Correlations between percent reduction in intact copies and percentage of CD3^-^CD56^+^ NK cells that stained positive for the indicated markers. Spearman’s r value shown.

Next, we tested whether PG16-Db and 3BNC117-Db mediated the elimination of HIV-1-infected cells under maximal latency reversal conditions ex vivo. We stimulated CD4^+^ T cells isolated from each of the 11 participants with PMA + I, cocultured them with autologous NK cells in the presence of PG16-Db, 3BNC117-Db or PG16-Db + 3BNC117-Db for 18 h, re-isolated annexin-V^−^CD4^+^ T cells and used the IPDA to measure the frequency of intact provirus^+^ cells. The frequency of intact provirus^+^ CD4^+^ T cells was significantly lower relative to the H2-Db control for five participants with PG16-Db, eight with 3BNC117-Db and nine with PG16-Db + 3BNC117-Db (Fig. 5a). Staining of untreated PBMCs from eight participants indicated no significant correlation between the reduction in the frequency of intact provirus^+^ CD4^+^ T cells and the expression of CD16, NKG2D, Siglec-7, CD57 or PD-1 (programmed cell death protein 1) on CD3^−^CD56^+^ NK cells (Extended Data Fig. 3), suggesting that the variability in response to scDb treatment did not correlate with NK cell phenotypic differences, at least as determined by these markers. Considering all participants, the PG16-Db, 3BNC117-Db and PG16-Db + 3BNC117-Db resulted in 41%, 45% and 49% average reductions in the frequency of intact provirus^+^ CD4^+^ T cells, respectively, when compared with H2-Db (Fig. 5a,b).

**Extended Data Fig. 4:**
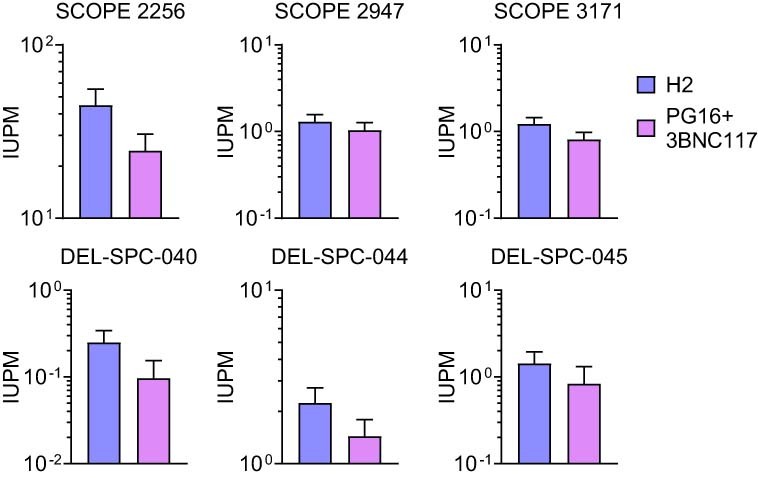
PG16-Db and 3BNC117-Db eliminate CD4^+^ T cells harboring inducible and replication-competent proviruses. CD4^+^ T cells were treated with PMA+I and co-culture with autologous NK cells in the presence of PG16-Db+3BNC117-Db. After co-culture, the viable CD4^+^ T cells were plated at limiting dilution for quantitative viral outgrowth assays (QVOAs). Concentrations of p24 was measured in the culture supernatants on day 14. Data reported as infectious units per million (IUPM) viable CD4^+^ T cells (maximum-likelihood estimate, 95% confidence interval upper bound).

Only a subset of intact provirus^+^ cells can be induced to produce virus with a single or multiple rounds of stimulation^30^. To better understand the effect of scDb treatment on the inducible population of reservoir cells, we plated the CD4^+^ T cells that survived coculture with autologous NK cells in the presence of PG16-Db and 3BNC117-Db at limiting dilution in quantitative viral outgrowth assays (QVOAs), which measure inducible, replication-competent proviruses. All six participants tested had reduced infectious units per million (IUPM) values when cocultures were treated with PG16-Db and 3BNC117-Db compared with H2-Db (Extended Data Fig. 4). PG16-Db + 3BNC117-Db resulted in a 40% reduction in IUPM on average compared with H2-Db (Fig. 5b).

**Extended Data Fig. 5:**
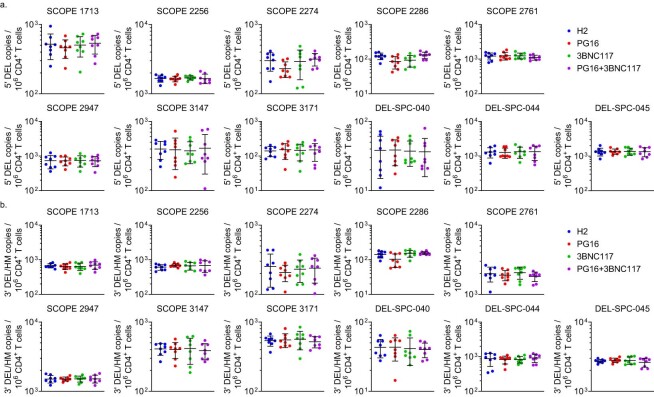
PG16-Db and 3BNC117-Db do not eliminate cells harboring defective proviruses *ex vivo*. Frequencies of CD4^+^ T cells harboring **a**, 5′ deleted or **b**, 3′ deleted or hypermutated proviruses after treatment with PMA+I and co-culture with autologous NK cells in the presence of PG16-Db, 3BNC117-Db, or PG16-Db+3BNC117-Db. Data reported as defective HIV-1 copies per million viable CD4^+^ T cells (mean, SD) from eight replicates.

The IPDA measures intact HIV-1 proviruses but also proviruses with common defects, including large internal deletions and hypermutation, which make up the vast majority of all proviruses in people with HIV-1 (refs. ^31,32^). Most defective proviruses are defective in the *tat* gene, thereby preventing high levels of viral gene expression^31,32^, and many have defects that affect the *env* gene, which prevent productive expression of Env protein and render the HIV-1-infected cells nondetectable by Env-specific antibodies. We observed no significant change in the frequency of CD4^+^ T cells with 5ʹ- or 3ʹ-defective proviruses in any of the 11 donors compared with H2-Db (Fig. 5b and Extended Data Fig. 5a,b), indicating that, in cultures in which intact provirus^+^ CD4^+^ T cells were eliminated, HIV-1-infected cells with highly defective proviruses were not affected, providing a compelling internal control. Thus, the observed reduction in the frequency of intact provirus^+^ CD4^+^ T cells was the result of scDb-mediated killing of HIV-1-infected cells that expressed high levels of Env. These results provide direct evidence that PG16-Db and 3BNC117-Db promote NK cell killing of latent reservoir cells from people on ART after ex vivo latency reversal.

### ScDb induces reduction of HIV-1^+^ cells in humanized mice

**Fig. 6:**
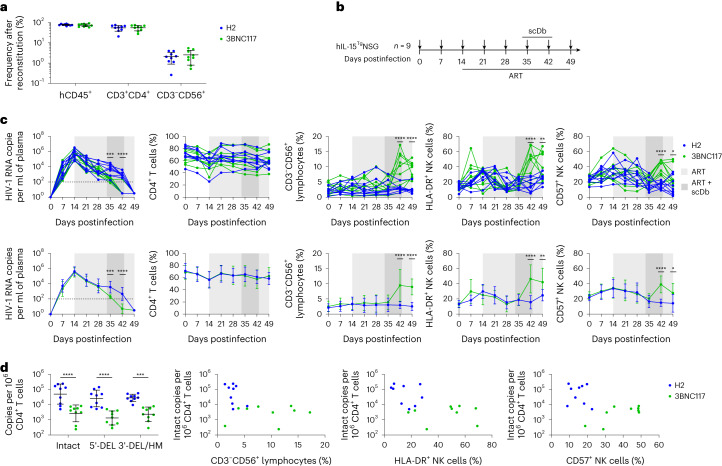
3BNC117-Db induces NK cell activity and elimination of infected cells in hIL-15^Tg^NSG mice. **a**, Frequencies of human CD4^+^ T cells and CD3^−^CD56^+^ NK cells in the peripheral blood at 12 weeks posttransfer of human fetal liver and thymus tissues into hIL-15^Tg^NSG mice. Mice were randomly assigned to two groups corresponding to treatment with H2-Db (*n* = 9) or 3BNC117-Db (*n* = 9) at day 33 postinfection. Data are reported as the percentage of PBMCs that expressed the indicated markers (mean, s.d.). **b**, Schematic showing hIL-15^Tg^NSG mice infected with HIV_SUMA_ at 12 weeks postreconstitution as in **a**, administered ART from day 14 to day 49 postinfection, injected intraperitoneally with H2-Db or 3BNC117-Db daily between day 33 and day 43 postinfection, sampled for peripheral blood on days 0, 7, 14, 21, 28, 35, 42 and 49 postinfection (small black arrows) and euthanized at day 49 postinfection for collection of splenic tissue. **c**, HIV-1 RNA copies per ml of plasma (far left), percentage of CD4^+^ T cells (middle left), percentage of CD3^−^CD56^+^ lymphocytes (middle), percentage of HLA-DR^+^ NK cells (middle right) and percentage of CD57^+^ NK cells (far right) from day 0 to day 49 postinfection of hIL-15^Tg^NSG mice. Data are shown as both individual values (top) and average values (bottom) for both the H2-Db- and 3BNC117-Db-treated mice (mean, s.d.): H2-Db (*n* = 9), 3BNC117-Db (*n* = 9). The significance was determined by two-way ANOVA followed by Šídák’s test for multiple comparisons: ^*^P < 0.05, ^**^P < 0.01, ^***^P < 0.001, ^****^P < 0.0001. **d**, Frequencies of intact provirus^+^ CD4^+^ T cells, 5′-deleted provirus^+^CD4^+^ T cells and 3′-deleted/hypermutated provirus^+^ CD4^+^ T cells in hIL-15^Tg^NSG mice at day 49 postinfection. Data are reported as HIV-1 copies per million human CD4^+^ T cells (mean, s.d.): H2-Db (*n* = 9), 3BNC117-Db (*n* = 9). The significance was determined by two-way ANOVA followed by Šídák’s test for multiple comparisons: ^***^P < 0.001, ^****^P < 0.0001. Comparisons between intact copies per million human CD4^+^ T cells percentage of CD3^−^CD56^+^ lymphocytes (middle left), percentage of HLA-DR^+^ NK cells (middle right) and percentage of CD57^+^ NK cells (far right) posttreatment (day 42).

**Extended Data Fig. 6:**
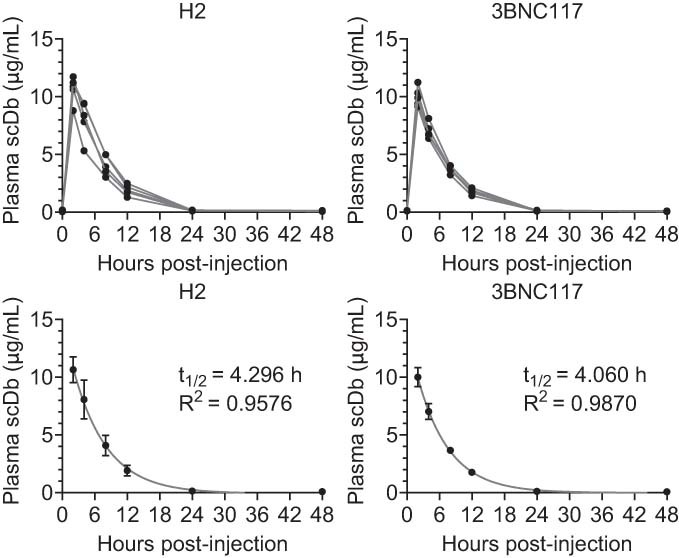
H2-Db and 3BNC117-Db plasma pharmacokinetics in hIL-15^Tg^NSG mice. The mice received a single 200 μg intraperitoneal injection of H2-Db or 3BNC117-Db and scDb plasma concentrations were assessed at 0, 2, 4, 8, 12, 24, and 48 hours post-injection. Plasma scDb concentrations are shown for the individual mice (top) or the average values (bottom) (mean, SD, 5 mice per group). Data were fitted to a single-phase exponential decay nonlinear regression model and predicted plasma half-lives are indicated.

**Extended Data Fig. 7:**
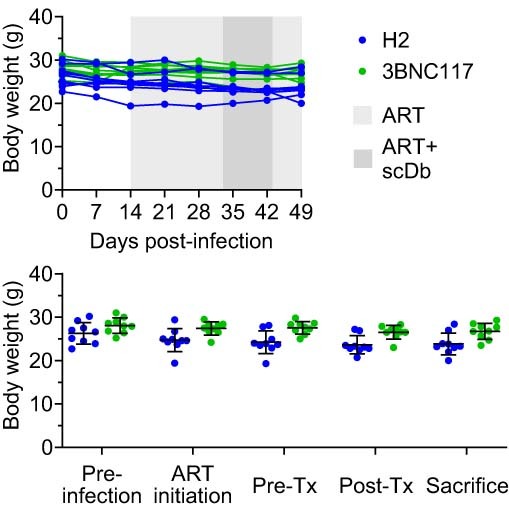
Tolerability of H2-Db and 3BNC117-Db in hIL-15^Tg^NSG mice. Body weight of the mice reported in grams. Data reported as weights of individual mice over time (top) and weights prior to infection, at the time of ART initiation, and before and after scDb treatment (bottom) (mean, SD). H2-Db (n = 9); 3BNC117-Db (n = 9). No significant differences between H2-Db- and 3BNC117-Db-treated mice at any time point or significant changes in body weights compared to day 0 within either group at any time point were determined by two-way ANOVA followed by Šídák’s test for multiple comparisons.

**Extended Data Fig. 8:**
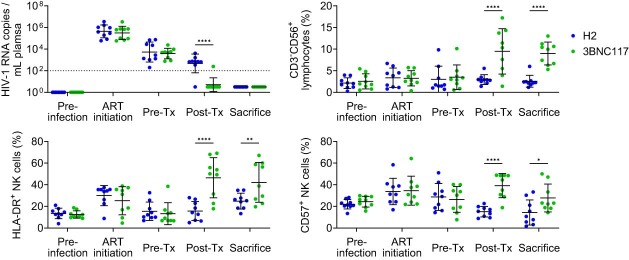
Additional measures of H2-Db and 3BNC117-Db-mediated activity in hIL-15^Tg^NSG mice. Plasma viremia and peripheral blood NK cell activity in the hIL-15^Tg^NSG mice prior to infection, at the time of ART initiation, and before and after H2-Db and 3BNC117-Db treatment. Data reported as HIV-1 RNA copies/mL plasma (top left), percentage of CD3^-^CD56^+^ lymphocytes (top right), percentage of HLA-DR^+^ NK cells (bottom left), and percentage of CD57^+^ NK cells (mean, SD). H2-Db (n = 9); 3BNC117-Db (n = 9). Significance determined by two-way ANOVA followed by Šídák’s test for multiple comparisons, *P < 0.05, **P < 0.01, ****P < 0.0001.

Next, we assessed the ability of one of the scDbs, 3BNC117-Db, to drive activation of NK cells and promote elimination of HIV-1-infected cells in humanized mice. Reconstitution of immunodeficient mice with human hematopoietic stem cells results in a reduced number of human NK cells with poor functional activity^33^. However, transgenic expression of human interleukin-15 (hIL-15) in mice restores the development of human NK cells with functional cytolytic and tissue-homing properties^34,35^. After reconstitution with hematopoietic stem cells, hIL-15 transgenic NOD.Cg-Prkdc^scid^Il2rg^tm1Wjl^ mice (hereafter called hIL-15^Tg^NSG) exhibited high frequencies of human CD4^+^ T cells and readily detectable human CD3^−^CD56^+^ NK cells (Fig. 6a). The hIL-15^Tg^NSG mice were infected with a transmitted/founder clone of HIV-1 (HIV_SUMA_), placed on ART to suppress viremia on day 14 postinfection, and administered 200 μg of H2-Db (*n* = 9) or 3BNC117-Db (*n* = 9) through intraperitoneal injection daily for 10 d starting on day 33 postinfection (Fig. 6b), a protocol selected based on prior evaluation of the plasma pharmacokinetics of the scDbs after a single injection (Extended Data Fig. 6). Mice were bled on days 0, 7, 14, 21, 28, 35, 42 and 49 postinfection to collect plasma and PBMCs, and were euthanized on day 49 postinfection for tissue collection. We did not observe evidence of toxicity or weight loss after scDb administration (Extended Data Fig. 7), suggesting that H2-Db and 3BNC117-Db were tolerated at the administered dose. Plasma viral load had reached an average of 3.67 × 10^5^ HIV-1 RNA copies per ml at the time of ART initiation (day 14 postinfection) and decayed to an average of 4.48 × 10^3^ HIV-1 RNA copies per ml by day 28 postinfection (Fig. 6c and Extended Data Fig. 8). As a result of the cytopathic effects of the virus during acute HIV-1 infection, the frequency of CD4^+^ T cells declined by 13% during the first 14 d postinfection (Fig. 6c). ART initiation resulted in recovery and stabilization of CD4^+^ T cell frequencies (Fig. 6c). No differences in plasma viral load or frequency of CD4^+^ T cells were detected between H2-Db- and 3BNC117-Db-treated mice at any timepoint before scDb administration. Treatment with 3BNC117-Db resulted in a faster decline of plasma HIV-1 RNA apparent at days 35 and 42 postinfection compared with H2-Db (Fig. 6c). By day 42, eight of nine mice that received 3BNC117-Db had attained suppression of plasma HIV-1 RNA to below the limit of detection compared with one of nine mice that received H2-Db (Fig. 6c and Extended Data Fig. 8).

**Extended Data Fig. 9:**
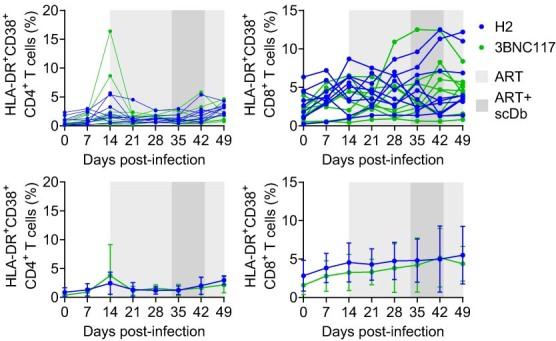
H2-Db and 3BNC117-Db do not induce global T cell activation in hIL-15^Tg^NSG mice. Frequency of peripheral blood CD4^+^ (left) and CD8^+^ (right) T cells that expressed HLA-DR and CD38 over time in the hIL-15^Tg^NSG mice. Data shown for individual mice (top) and average percentages (bottom) (mean, SD). H2-Db (n = 9); 3BNC117-Db (n = 9). No significant differences between H2-Db- and 3BNC117-Db-treated mice at any time point or significant changes in frequencies of HLA-DR^+^CD38^+^ T cells compared to day 0 within either group at any time point were determined by two-way ANOVA followed by Šídák’s test for multiple comparisons.

We then examined the effect of 3BNC117-Db on peripheral blood NK cells by quantifying the expansion of total CD3^−^CD56^+^ NK cells within the CD45^+^ lymphocytes and the expression of human leukocyte antigen (HLA)-DR, an activation marker, and CD57, an NK cell maturation marker associated with superior NK cell cytolytic and cytokine-secreting functions^36^. The frequencies of HLA-DR^+^ NK cells and CD57^+^ NK cells increased over the first 14 d postinfection (Fig. 6c and Extended Data Fig. 8), probably corresponding to the emergence of NK cell responses against HIV-1 during acute infection. The increase in HLA-DR^+^ NK cells and CD57^+^ NK cells was transient, and their frequencies declined to near baseline levels after ART initiation (Fig. 6c and Extended Data Fig. 8). After scDb administration, the frequency of peripheral blood CD3^−^CD56^+^ NK cells was on average 3.2-fold higher in mice that received 3BNC117-Db compared with mice that received H2-Db at day 42 postinfection (Fig. 6c and Extended Data Fig. 8), whereas the frequencies of HLA-DR^+^ NK cells and CD57^+^ NK cells increased 3.0-fold and 2.6-fold on average, respectively, in mice that received 3BNC117-Db compared with mice that received H2-Db at day 42 postinfection (Fig. 6c). The increased frequencies of total CD3^−^CD56^+^ NK cells, HLA-DR^+^ NK cells and CD57^+^ NK cells in the 3BNC117-Db-treated mice waned slightly after cessation of scDb administration at day 43 postinfection, but remained significantly elevated compared with H2-Db-treated mice at day 49 (Fig. 6c and Extended Data Fig. 8). We found no significant differences in the frequencies of CD4^+^ or CD8^+^ T cells that expressed the activation markers HLA-DR or CD38 during or after scDb administration in 3BNC117-Db- and H2-Db-treated mice (Extended Data Fig. 9), indicating that 3BNC117-Db did not result in global T cell activation.

**Extended Data Fig. 10:**
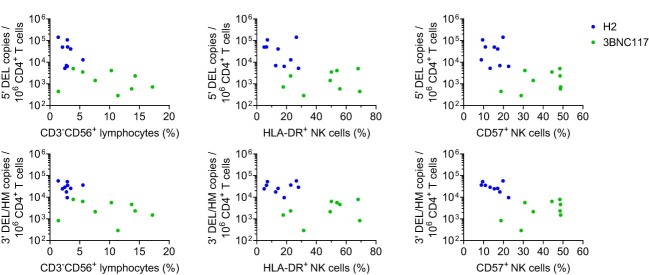
Comparisons between the frequencies of defective provirus^+^CD4^+^ T cells and NK cell activity in H2-Db- and 3BNC117-Db-treated hIL-15^Tg^NSG mice. Data reported as the percentage of CD3^-^CD56^+^ lymphocytes (left), percentage of HLA-DR^+^ NK cells (middle), and percentage of CD57^+^ NK cells (right) post-treatment (day 42) compared to the frequencies of CD4^+^ T cells harboring 5′ deleted (top) or 3′ deleted or hypermutated proviruses (bottom). H2-Db (n = 9); 3BNC117-Db (n = 9).

Last, IPDA quantification of HIV-1 reservoir size on DNA extracted from splenocytes at day 49 postinfection, when all mice had reached undetectable plasma HIV-1 RNA levels, showed that 3BNC117-Db-treated mice had a 25-fold average reduction in the frequency of intact provirus^+^ CD4^+^ T cells, a 23-fold average reduction in the frequency of CD4^+^ T cells harboring proviruses with 5′-deletion and a 9-fold average reduction in CD4^+^ T cells harboring proviruses with 3′-deletion or hypermutation compared with H2-Db-treated mice (Fig. 6d). Mice treated with 3BNC117-Db also exhibited increased frequencies of CD3^−^CD56^+^ NK cells, and higher fractions of HLA-DR^+^ NK cells and CD57^+^ NK cells posttreatment (day 42) relative to mice treated with H2-Db (Fig. 6d and Extended Data Fig. 10). Thus, 3BNC117-Db promoted enhanced NK cell-mediated elimination of infected CD4^+^ T cells, which resulted in a smaller reservoir size at the time of complete ART-mediated viral suppression.

## Discussion

In the present study, we developed two bispecific antibodies that target Env and CD16 to enhance HIV-1-specific NK cell activity. PG16-Db and 3BNC117-Db promoted highly potent and specific NK cell activation and NK cell-mediated killing of HIV-1-infected cells. These bispecific antibodies mediated the elimination of up to 72% of intact provirus^+^ CD4^+^ T cells from people on suppressive ART ex vivo, thus representing promising new therapeutics for HIV-1 reservoir reduction strategies. In addition, our evaluation of 3BNC117-Db in HIV-1-infected humanized mice suggested that bispecific antibodies may also have potential application for use in limiting reservoir establishment during acute HIV-1 infection or reducing reservoir size during chronic HIV-1 infection.

By using a high-affinity CD16 scFv, we developed scDbs that elicit stronger NK cell activation and killing of infected cells compared with parental IgG bNAbs. PG16-Db and 3BNC117-Db elicited HIV-1-specific NK cell activity and elimination of HIV-1-infected CD4^+^ T cells in vitro, at levels comparable to those of previously reported Env-CD16-bispecific antibodies^37,38^. However, unlike previously reported Env-CD16-bispecific antibodies, the scDbs described in the present study were not dependent on prestimulation of the NK cell effectors with IL-2 or IL-15. In addition, scDb binding to CD16 alone did not result in notable NK cell activation or cytolysis of uninfected cells, an important consideration for in vivo applications. ScDb-induced activation of other CD16-expressing immune effectors such as neutrophils, macrophages and γδ T cells may occur, representing a possible safety concern in vivo, but also potentially allowing for further enhancement of infected CD4^+^ T cell clearance.

Ongoing viral replication reduces the fraction of cytotoxic CD56^dim^CD16^+^ NK cells and promotes the emergence of dysfunctional NK cell responses^39,40^. However, ART can result in a partial or complete restoration of the CD56^dim^CD16^+^ NK cell subset and NK cell activity^39,41,42^. We did not find a relationship between scDb-enhanced NK cell cytotoxicity ex vivo and NK cell phenotype in the ART-treated people studied here.

The present study and others^29^ have shown that single LRA treatments cause only small increases in HIV-1 caRNA transcripts. Consequently, none of the individual LRAs tested was probably able to induce high levels of Env expression in most intact provirus^+^ cells, thereby limiting elimination of those cells when paired with autologous NK cell coculture. Env surface density is a determinant of infected cell elimination for other Env-specific immunotherapeutic approaches, including bNAb-based CAR T cells^43^. To achieve robust elimination of reservoir cells in vivo with Env-directed therapeutics, single or combinations of LRAs with greater potency will be needed.

The efficient elimination of intact provirus^+^ cells across multiple participants suggested that PMA + I treatment resulted in sufficient Env expression for scDb-mediated elimination. We did not observe a reduction in cells that harbored proviruses with deletions in *env* or extensive hypermutation in any participant, because such cells are not able to express Env. Cells that harbored proviruses with 5′-deletions also escaped elimination. Many of these proviruses have defects that affect the major splice donor site (MSD)^31,32^. As the mRNA encoding Env is generated by a splicing reaction involving the MSD site, MSD defects markedly reduce Env expression^44^ and may prevent targeting by scDbs.

By combining PG16-Db and 3BNC117-Db, which target nonoverlapping Env epitopes, we observed a slightly greater average elimination of reservoir cells than either single scDb mediated at the same concentration. This may reflect either the improved breadth of viral variant recognition attainable with multiple bNAbs or synergy due to simultaneous binding to multiple epitopes on Env. To achieve more potent and universally effective elimination of reservoir cells, future efforts should explore combinations of targeting modalities. The scDbs described in the present study may complement CD8^+^ T cell-based bispecific antibodies^14–16^ by engaging multiple cytolytic immune cell types. In addition, bispecific antibodies that target HIV-1 peptide-major histocompatibility complexes^16^ may synergize with Env-targeted approaches by enabling the elimination of reservoir cells with suboptimal Env expression.

A limitation of our humanized mouse model is that the reduction in infected CD4^+^ T cells probably reflects the elimination of productively infected CD4^+^ T cells, which comprise most of the infected cell population during the initial stages of HIV-1 infection rather than the elimination of latently infected CD4^+^ T cells. Future nonhuman primate studies, in which a combination treatment of scDbs and LRAs is administered in the context of long-term viral suppression, will be necessary to directly evaluate scDb-mediated clearance of latent reservoir cells. Despite this caveat, these findings suggest that scDbs may have potential clinical applications during acute HIV-1 infection at the time of ART initiation. NK cell functional activity during acute HIV-1 infection correlates with viral control^45,46^ and less severe disease progression^47^ during chronic infection. High frequencies of CD57^+^ NK cells during acute HIV-1 infection have been associated with lower viral load after 3 months on ART and faster time to viral suppression^48^. In addition, the CD57^+^ NK cell subset exhibits highly potent ADCC and cytokine-producing activity in people with HIV-1 (ref. ^36^). Thus, the potential for 3BNC117-Db to promote elimination of reservoir cells during early HIV-1 infection warrants further exploration.

The short half-life of the scDbs in humanized mice probably resulted from the absence of Fc domains which, through interaction with the neonatal Fc receptor, extended the half-life of traditional monoclonal IgG-based therapeutics. The fast decay rate of scDbs in plasma may be a challenge for clinical application, because daily injections or continuous infusion will probably be necessary to maintain therapeutic concentrations. ScDbs may benefit from advances in protein engineering aimed at extending the half-life of small recombinant antibody molecules in vivo^49,50^. In addition, as the scDbs are engineered constructs with features that do not resemble naturally occurring antibodies, the emergence of scDb-specific antibody responses that could affect clinical treatment efficacy may occur. Although we did not find evidence of induction of global immune activation, a detailed profiling of cytokine responses induced by administration of scDbs in vivo will be necessary to alleviate safety concerns over the potential for cytokine-related adverse reactions.

Taken together, PG16-Db and 3BNC117-Db deserve additional evaluation as therapeutic interventions for HIV-1 reservoir reduction. We expect future studies to focus on combining these scDbs with other treatments to further improve on attainable clearance of the HIV-1 reservoir cells.

## Methods

### Human samples

Leukapheresis samples from people on suppressive ART were obtained from the University of Pennsylvania (BEAT-HIV Delaney cohort, DEL-SPC) and the University of California San Francisco (SCOPE cohort). Selection criteria included undetectable plasma HIV-1 RNA levels (<50 copies per ml) for >72 months. In total, samples from 11 male HIV^+^ participants were collected (median age = 56 years, range = 34–69 years; median time on ART = 289 months, range = 130–360 months; median time on suppressive ART = 185 months, range = 79−275 months; see Supplementary Table 2 for ART regimens). This protocol was approved by the institutional review boards at the University of Pennsylvania and the University of California San Francisco. All participants provided written informed consent. Leukapheresis samples from three HIV^−^ participants (two women and one man, aged 29, 46 and 48 years) were obtained from Stemcell Technologies. PBMCs were purified from leukapheresis samples by density gradient centrifugation with Ficoll Paque Plus (GE Healthcare) and cryopreserved.

### ScDb production

The gBlocks (IDT) encoding the scDb, an amino-terminal IL-2 signal sequence and a carboxy-terminal 6× His tag were cloned into the pcDNA3.4 vector (Thermo Fisher Scientific). ScDbs were then transiently expressed in Expi293 cells by GeneArt (Thermo Fisher Scientific) and purified from culture supernatant by HisTrap column (GE Healthcare), followed by size exclusion chromatography with a HiLoad Superdex 200 16/600 column (GE Healthcare). Analytical chromatography was performed using a TSKgel G3000SWxl column (TOSOH Bioscience) and a running buffer of 50 mM sodium phosphate and 300 mM sodium chloride, pH 7, at a flow rate of 1.0 ml min^−1^.

### NK cell-surface CD16-binding assay

NK and B cells were isolated from uninfected participant PBMCs (Stemcell Technologies), plated in BCM (RPMI 1640 with 10% fetal bovine serum (FBS) and 1% penicillin–streptomycin) with or without CD16 mIgG-blocking antibody (BioLegend, catalog no. 302001, 1 μg ml^−1^), and incubated at 37 °C for 1 h. The cells were washed in phosphate-buffered saline (PBS) and stained with scDbs (at indicated concentrations, in PBS) at 4 °C for 1 h. The cells were washed and stained with the following antibodies (1:50 dilution)/dye (1:1,000 dilution) at 4 °C for 45 min: CD3-BV605, CD19-BV421, CD56-PE/Cy5 (BioLegend, catalog nos. 317321, 302233 and 304607, respectively), 6× His tag-AF488 and Fixable Viability Dye-eFluor780 (Thermo Fisher Scientific, catalog nos, MA1-135-A488 and 65-0865-14, respectively). The cells were washed, and samples were acquired on an Intellicyt iQue Screener Plus (Sartorius). Data were analyzed with FlowJo (BD Bioscience; see Supplementary Fig. 4 for gating example). NK cells were defined as live CD3^−^CD19^−^CD56^+^ lymphocytes, T cells as live CD3^+^ lymphocytes and B cells as live CD19^+^ lymphocytes. All conditions were tested in triplicate, mean and s.d. shown.

### CD16-binding ELISA

CD16–biotin recombinant proteins (F/V158, Acrobiosystems, 0.5 μg ml^−1^) in BAE blocking buffer (PBS, 0.5% bovine serum albumin, 0.1% sodium azide) were added to an EvenCoat streptavidin-coated plate (R&D Systems) and incubated at 4 °C for 16 h. The plate was washed with 1× TBS-T (1× tris-buffered saline (TBS) + 0.05% Tween-20) using a BioTek 405 plate washer and scDbs (at indicated concentrations, in BAE) were plated and incubated at 4 °C for 16 h. The plate was washed as before and HRP (horse radish peroxidase) Protein L secondary (Thermo Fisher Scientific, catalog no. 32420, 0.5 μg ml^−1^ in BAE) was plated and incubated at room temperature for 1 h. The plate was washed and developed in TMB (3,3′,5,5′-tetramethylbenzidine) for 5 min at room temperature before stopping in 1 N sulfuric acid. The optical density at 450 nm (OD_450_) was read; all conditions were tested in triplicate, mean and s.d. shown.

### Surface Env-binding assay

HEK293T cells (transformed embryonic kidney cells, American Type Culture Collection, catalog no. CRL-3216) were transfected with a panel of HIV-1 *env* expression plasmids. Then, 3 d posttransfection, transfected HEK293T cells were stained with scDbs (at indicated concentrations, in Dulbecco’s modified Eagle’s medium (DMEM) with 10% FBS and 1% penicillin–streptomycin) at 37 °C for 1 h. The cells were washed in PBS and stained with the following secondary antibody/dye at 4 °C for 45 min: 6× His tag-AF488 and Fixable Viability Dye-eFluor780. The cells were washed, and samples were acquired on an Intellicyt iQue Screener Plus. Data were analyzed with FlowJo (see Supplementary Fig. 5 for gating example). In addition, a subset of transfected HEK293T cells were stained with the parental IgG molecules or an IgG1 isotype control (at 20 μg ml^−1^ in DMEM with 10% FBS and 1% penicillin–streptomycin) to allow for comparison of Env expression. The cells were washed in PBS and stained with the following secondary antibodies (1:50 dilution)/dye (1:1,000 dilution) at 4 °C for 45 min: IgG Fc-BV421 (BioLegend; catalog no. 409318) and Fixable Viability Dye-eFluor780. The cells were washed and samples were acquired on an Intellicyt iQue Screener Plus. Data were analyzed with FlowJo. All conditions were tested in triplicate, mean and s.d. shown.

### NK activation coculture

A3.01 and ACH-2 cells (transformed CD4^+^ T cells, National Institutes of Health (NIH) AIDS Reagents Program, catalog nos. ARP-166 and ARP-349, respectively) were plated in BCM with 10 ng ml^−1^ of TNF and incubated at 37 °C for 18 h. The cells were washed in BCM to remove TNF. NK cells were isolated from uninfected participant PBMC and cocultured with either the reactivated A3.01 or ACH-2 cells at a 1:3 E:T ratio in BCM at 37 °C for 6 h in the presence of the scDbs or the parental IgG molecules (at the indicated concentrations), monensin (BD Bioscience) and CD107a-BV421 (BioLegend, catalog no. 328625). The supernatants were collected and analyzed by Human CD8/NK Panel Legendplex (BioLegend) per the manufacturer’s protocol. The cells were washed in PBS and stained with the following antibodies (1:50 dilution)/dye (1:1,000 dilution) at 4 °C for 45 min: CD3-BV605 and CD56-FITC (BioLegend; catalog nos. 317321 and 304603, respectively) and Fixable Viability Dye-eFluor780. The cells were washed, and samples were acquired on an Intellicyt iQue Screener Plus. Data were analyzed with FlowJo (see Supplementary Fig. 6 for gating example). NK cells are defined as live CD3^−^CD56^+^ lymphocytes. All conditions were tested in triplicate, mean and s.d. shown.

### In vitro infected cell elimination coculture

CD4^+^ T cells were isolated from uninfected participant PBMCs, plated in BCM with IL-2 (30 U ml^−1^) and CD3/CD28 Dynabeads (Thermo Fisher Scientific) and incubated at 37 °C for 3 d. The activated CD4^+^ T cells were spinoculated with replication-competent NL4.3-ΔNef-eGFP at 100 ng of p24 per 100,000 cells (in conditioned antiretrovirals (ARVs; 10 μM tenofovir disoproxil fumarate, 10 μM emtricitabine and 10 μM dolutegravir)) were added to the infected cells for 12 h to stop ongoing infection. NK cells were isolated from autologous PBMCs and cocultured with the infected CD4s at a 1:3 E:T ratio in BCM with ARVs at 37 °C for 18 h in the presence of the scDbs or their parental IgG molecules (at indicated concentrations). The cells were washed in PBS and stained with the following antibodies (1:50 dilution)/dye (1:1,000) at 4 °C for 45 min: CD3-BV421, CD56-PE/Cy5 (BioLegend; catalog nos. 317343 and 304607, respectively) and Fixable Viability Dye-eFluor780. The cells were washed and samples were acquired on an Intellicyt iQue Screener Plus. Data were analyzed with FlowJo (see Supplementary Fig. 7 for gating example). CD4^+^ T cells were defined as live CD3^+^CD56^−^ lymphocytes. Supernatant p24 measurements were performed using the Alliance HIV-1 p24 Antigen ELISA kit (PerkinElmer) according to the manufacturer’s protocol. Spinoculation of activated CD4^+^ T cells with replication-competent NL4.3 and subsequent coculture with autologous NK cells and staining were performed as above, except after the initial surface staining; the cells were permeabilized using the Fixation/Permeabilization Kit (BD Bioscience) and stained with p24-FITC (Abcam, catalog no. ab20569, 1:50 dilution) at 4 °C for 45 min before the final wash in PBS and acquisition. Before coculture, a subset of NL4.3-ΔNef-eGFP-infected CD4^+^ T cells was stained with the parental IgG molecules or an IgG1 isotype control (at 20 μg ml^−1^ in conditioned T cell medium) to measure Env expression. After a 1-h incubation at 37 °C, the cells were washed in PBS and stained with the following antibodies (1:50 dilution)/dye (1:1,000) at 4 °C for 45 min: CD3-BV605, human IgG Fc-BV421 (BioLegend; catalog nos. 317321 and 409318, respectively) and Fixable Viability Dye-eFluor780. The cells were washed and samples were acquired on an Intellicyt iQue Screener Plus. Data were analyzed with FlowJo. CD4^+^ T cells were defined as live CD3^+^ lymphocytes. All conditions were tested in triplicate, mean and s.d. shown.

### Ex vivo elimination of latency reversed cell coculture

CD4^+^ T cells were isolated from PBMCs of participants on suppressive ART (Supplementary Table 2) and plated in BCM with a stimulating agent and ARVs (10 μM tenofovir disoproxil fumarate, 10 μM emtricitabine and 10 μM dolutegravir) and incubated at 37 °C for 18 h. Stimulating agents were added as follows: bryostatin (10 nM), AZD5582 (500 nM), SAHA (335 nM), romidepsin (40 nM), CD3/CD28 (Dynabeads, Thermo Fisher Scientific, according to the manufacturer’s protocol), PMA (50 ng ml^−1^) and ionomycin (1 μM). The CD4^+^ T cells were washed in BCM with ARVs to remove the PMA and ionomycin. NK cells were isolated from autologous PBMCs and cocultured with the infected CD4s at a 1:3 E:T ratio in BCM with ARVs at 37 °C for 18 h in the presence of the scDbs (200 pM). CD4^+^ T cells were re-isolated from the cocultures and depleted of apoptotic cells by annexin-V immunomagnetic selection. DNA isolation and quantification of HIV-1 DNA by IPDA and RPP30 cell equivalents were performed as previously described^51,52^. IPDA measurements were performed in replicates of eight, individual values, mean and s.d. shown. Viable CD4^+^ T cells were plated at a limiting dilution for QVOAs, which were performed as previously described^53^, no supernatants were harvested for p24 measurement at day 14. Before coculture, NK cells from a subset of study participants were stained to evaluate surface expression of CD16, NKG2D, Siglec-7, CD57 and PD-1. The cells were incubated with the following antibodies (1:50 dilution)/dye (1:1,000) at 4 °C for 45 min in PBS: CD3-BV605 and CD56-FITC, Fixable Viability Dye-eFluor780 and CD16-BV421, NKG2D-BV421, Siglec-7-PerCP/Cy5.5, CD57-PerCP/Cy5.5 or PD-1-PE/Cy5 (BioLegend; catalog nos. 302038, 320822, 339216, 359622 or 329971, respectively). The cells were washed and samples were acquired on an Intellicyt iQue Screener Plus. Data were analyzed with FlowJo. NK cells were defined as live CD3^−^CD56^+^ lymphocytes. All conditions were tested in triplicate, mean and s.d. shown.

### Cell-associated polyadenylated HIV-1 RNA measurement

Isolation of caRNA was performed as previously described^54,55^. Oligo(dT) complementary DNA synthesis was performed using Superscript III Reverse Transcriptase (Thermo Fisher Scientific) previously published methods^54,55^. All conditions were tested in triplicate, mean and s.d. shown.

### Generation of the hIL-15^Tg^NSG mice

The hIL-15^Tg^NSG mice were generated as previously described^56–58^. All mice experiments were approved by the Wistar Institute Animal Care and Research Committee (protocol no. 201360). All animals recruited in the present study were housed in the Wistar Institute humanized mice holding room with a 12-h light:dark cycle at temperatures of 20–23 °C and 40–60% humidity. Briefly, 6- to 8-week-old female NSG-Tg(hIL-15) (NOD.Cg-Prkdc^scid^ Il2rg^tm1Wjl^ Tg(IL15)1Sz/SzJ; Jackson Laboratory^59^) mice were pretreated with busulfan at 30 mg kg^−1^ and were then implanted with human fetal thymic tissue fragments and fetal liver tissue fragments under the murine renal capsule. After the surgery, mice were injected via the tail vein with CD34^+^ hematopoietic stem cells isolated from human fetal liver tissues. Human fetal liver and thymus tissues were procured from Advanced Bioscience Resources. Then 12 weeks postsurgery, human immune cell reconstitution in peripheral blood was determined using a FACSymphony flow cytometer (BD Biosciences) using the following antibodies (1:50 dilution): mCD45-AF700, hCD45-FITC, hCD3-BUV805, hCD4-BUV395, hCD8-PerCP-Cy5.5, hCD56-BV650 and Fixable Viability Stain 510 (catalog nos. 560510, 555482, 612895, 563550, 565310, 564057 and 564406, respectively; BD Biosciences). Data were analyzed with FlowJo.

### HIV-1 infection, ART suppression and scDb administration of the hIL-15^Tg^NSG mice

The hIL-15^Tg^ NSG mice were randomly divided into two groups (*n* = 9) and were infected intravenously with 1 × 10^4^ TCID_50_ (50% tissue culture infectious dose) of T/F virus HIV_SUMA_. Peripheral blood was collected weekly for plasma viral load assays. Then 2 weeks postinfection, the mice were placed on a diet combined with ART until the end of the study (1,500 mg kg^−1^ of emtricitabine, 1,560 mg kg^−1^ of tenofovir disoproxil fumarate and 600 mg kg^−1^ of raltegravir). Beginning on day 33 and continuing through day 43 postinfection, the mice were given daily intraperitoneal injections of 200 μg (approximately 7 mg kg^−1^) of either H2-Db or 3BNC117-Db. The mice were euthanized at 7 weeks postinfection and blood and tissues were collected.

### Plasma viral load measurement

Plasma viral loads were measured as previously described^56–58,60,61^. Briefly, viral RNA was extracted using a QIAamp Viral RNA Mini kit (QIAGEN). The pVLs were determined using reverse transcription quantitative PCR (RT-qPCR) on a C1000 Thermal Cycler and the CFX96 Real-Time system (BioRad) with the TaqMan Fast Virus 1-Step Master Mix (Life Technologies).

### Measuring markers of T cell and NK cell activation and maturation

Peripheral blood and spleen cell suspensions were collected weekly or at the end of study, respectively, for flow cytometry analysis. Cells were stained using the following antibodies (1:50 dilution): mCD45-AF700, hCD45-FITC, hCD3-BUV805, hCD4-BUV395, hCD8-PerCP-Cy5.5, hCD56-BV650, HLA-DR-APC, hCD38-PE, hCD57-PE-CF594 and Fixable Viability Stain 510 (catalog nos. 560510, 555482, 612895, 563550, 565310, 564057, 340691, 555460, 562488 and 564406, respectively; BD Biosciences). Data were collected on a FACSymphony flow cytometer (BD Biosciences).

### The IPDA in hIL-15^Tg^NSG mice

A single-cell suspension of splenocytes was generated using the gentleMACS Octo Dissociator (Miltenyi Biotec). Genomic DNA was extracted from the splenocytes of the IL-15 Tg BLT mice. Each IPDA ddPCR reaction was performed in parallel with a copy reference/shearing correction (*RPP30* gene) ddPCR reaction which quantifies input human cell equivalents and DNA shearing^51^. Input human CD4^+^ T cell equivalents were calculated based on flow cytometry staining of the splenocyte samples as follows: (Input human RPP30 cell equivalents) × (percentage hCD4^+^/hCD45^+^ cells). Shearing corrections were applied to the IPDA data and the final results are reported as copies per 10^6^ human CD4^+^ T cells.

### Statistics and reproducibility

Sample sizes are noted for each experiment in the relevant figure legends. No statistical method was used to predetermine sample sizes; they were determined based on availability of biological samples. Controls were included as appropriate to provide a reference with which to compare experimental data. No data were excluded from any analyses. For all in vitro and ex vivo culture experiments, all conditions were tested for each participant in parallel. Assignment of hIL-15^Tg^NSG mice to treatment groups was randomized. During data collection and analysis for all in vitro and ex vivo culture experiments and for hIL-15^Tg^NSG mice-related experiments, the investigators were blinded to the treatment conditions of the relevant biological samples. Statistical tests used in the present study included analysis of variance (ANOVA) with multiple comparisons (Figs. 1b, 2a,c, 3b, 4a,b, 5a,b and 6c,d, Extended Data Figs. 2c,d and 7–9 and Supplementary Fig. 3), Student’s *t*-tests (Figs. 1d and 5b), Spearman’s correlations (Fig. 4c and Extended Data Fig. 3) and nonlinear regression models (Extended Data Figs. 1a and 6). All statistical analyses were performed using GraphPad Prism. Data distribution was assumed to be normal but this was not formally tested. *P* < 0.05 was considered significant for all tests. For all figures, ^*^P < 0.05, ^**^P < 0.01, ^***^P < 0.001 and ^****^P < 0.0001

### Reporting summary

Further information on research design is available in the Nature Portfolio Reporting Summary linked to this article.

## Online content

Any methods, additional references, Nature Portfolio reporting summaries, source data, extended data, supplementary information, acknowledgements, peer review information; details of author contributions and competing interests; and statements of data and code availability are available at 10.1038/s41590-023-01741-5.

### Supplementary information

Supplementary Information Supplementary Tables 1 and 2, Figs. 1–7 and all associated legends.

Reporting Summary

## Data Availability

Data that support the findings of the present study are available upon request via email to the lead corresponding author R.F.S. (rsiliciano@jhmi.edu). Data involving human research participants are subject to the data protection constraints in the written informed consent signed by the study participants.
