# Supplementary material for: Bispecific antibodies promote natural killer cell-mediated elimination of HIV-1 reservoir cells
Source: Nat Immunol. 2024 Jan 26;25(3):462–70. doi: 10.1038/s41590-023-01741-5 (PMC10907297; doi:10.1038/s41590-023-01741-5)
Supplement: Supplementary file 1 — Supplementary Tables 1 and 2, Figs. 1–7 and all associated legends. [file 41590_2023_1741_MOESM1_ESM.pdf]

nature immunology

Article

https://doi.org/10.1038/s41590-023-01741-5

Bispecific antibodies promote natural killer cell-mediated elimination of HIV-1 reservoir cells

In the format provided by the
authors and unedited

scFv	antigen	V_H	V_L
H2	human p53^{R175H} pHLA-A2	EVQLVESGGGLVQPGGSLRLSCA<br>ASGFNVYASGMHWVRQAPGKGL<br>EWVAKIYPDSDYTYYADSVKGRFT<br>ISADTSKNTAYLQMNSLRAEDTAV<br>YYCSRDSSFYYVYAMDYWGQGTL<br>VTVSS	DIQMTQSPSSLSASVGDRVTITCR<br>ASQDVNTAVAWYQQKPGKAPKLLI<br>YSAYFLYSGVPSRFSGSRSGTDFT<br>LTISSLQPEDFATYYCQQYSRYSP<br>VTFGQGTKVEIK
PG16	HIV-1 Env V1/V2	QEQLVESGGGVVQPGGSLRLSCL<br>ASGFTFHKYGMHWVRQAPGKGLE<br>WVALISDDGMRKYHSDSMWGRVT<br>ISRDNSKNTLYLQFSSLKVEDTAM<br>FFCAREAGGPIWHDDVKYYDFND<br>GYYNYHYMDVWGKGTTVTVSS	QSALTQPASVSGSPGQTITISCNG<br>TSSDVGGFDSVSWYQQSPGKAPK<br>VMVFDVSHRPSGISNRFSGSKSG<br>NTASLTISGLHIEDEGDYFCSSLTD<br>RSHRIFGGGTKVTVL
3BNC117	HIV-1 Env CD4bs	QVQLLQSGAAVTKPGASVRVSCE<br>ASGYNIRDYFIHWWRQAPGQGLQ<br>WVGWINPKTGQPNNPRQFQGRV<br>SLTRHASWDFDTFSFYMDLKALRS<br>DDTAVYFCARQRSDYWDFDVWG<br>SGTQVTVSS	DIQMTQSPSSLSASVGDTVTITCQ<br>ANGYLNWYQQRRGKAPKLLIYDG<br>SKLERGVPSRFSGRRWGQEYNLT<br>INNLQPEDIATYFCQVYEFVVPGTR<br>LDLKRTVA
NM3E2	human CD16	EVQLVESGGGVVRPGGSLRLSCA<br>ASGFTFDDYGMSWVRQAPGKGLE<br>WVSGINWNGGSTGYADSVKGRFT<br>ISRDNAKNSLYLQMNSLRAEDTAV<br>YYCARGRSLLFDYWGQGTLVTVS<br>R	SSELTQDPAVSVALGQTVRITCQG<br>DSLRSYYASWYQQKPGQAPVLVIY<br>GKNNRPSGIPDRFSGSSSGNTASL<br>TITGAQAEDEADYYCNSRDSSGN<br>HVVFGGGTKLTVG

Supplementary Table 1: Sequences of the V_H and V_L regions for the relevant scDbs.

Participant ID	Age	Sex	Race	Time since diagnosis (months)	ART regimen	Time on ART (months)	Time on suppressive ART (months)
SCOPE 1713	37	M	White	130	FTC/TAF/BIC	130	124
SCOPE 2256	66	M	White	437	FTC/TAF/RPV/TCV	347	248
SCOPE 2274	60	M	White	235	FTC/TAF/NVP	222	220
SCOPE 2286	56	M	White	233	FTC/TAF/RPV	233	229
SCOPE 2761	49	M	Hispanic	289	FTC/TAF/BIC	289	275
SCOPE 2947	56	M	Hispanic	368	3TC/ABC/TDF/NVP	344	79
SCOPE 3147	65	M	Hispanic	342	FTC/TAF/BIC	320	172
SCOPE 3171	69	M	Mixed	329	FTC/TAF/RGV/DRV/RTV	326	193
DEL-SPC-040	34	M	Mixed	153	3TC/ABC/TCV	146	135
DEL-SPC-044	55	M	AA	238	FTC/TAF/BIC	216	176
DEL-SPC-045	68	M	White	384	FTC/TDF/ATV/RTV	360	86

Supplementary Table 2: Characteristics of the study participants. Abacavir, ABC; African American, AA; antiretroviral therapy, ART; atazanavir, ATV; bictegravir, BIC; darunavir, DRV; dolutegravir, TCV; emtricitabine, FTC; lamivudine, 3TC; nevirapine, NVP; raltegravir, RGV; rilpivirine, RPV; ritonavir, RTV; tenofovir alafenamide, TAF; tenofovir disoproxil fumarate, TDF.

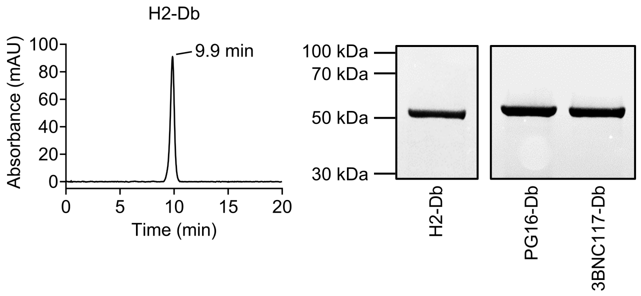

Supplementary Figure 1: H2-Db, PG16-Db, and 3BNC117-Db purity after size-exclusion chromatography. Representative analytical chromatogram of the purified H2-Db (left). Data reported as absorbance at 280 nm over retention time. The peak retention time is indicated above the chromatogram. SDS-PAGE analysis of H2-Db, PG16-Db, and 3BNC117-Db (right). Expected molecular weights of H2-Db, PG16-Db, and 3BNC117-Db are 52 kDa, 54 kDa, and 53 kDa respectively.

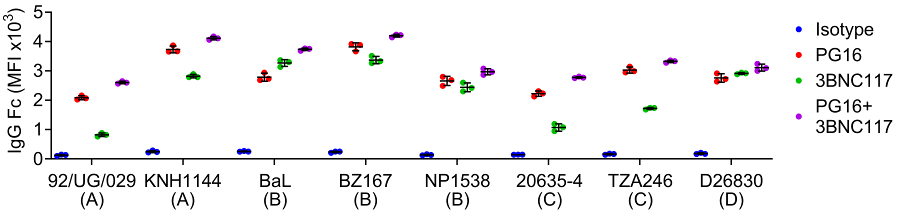

Supplementary Figure 2: Expression of Env on transfected HEK293T cells. Binding of PG16-Ig and 3BNC117-Ig to HEK293T cells transfected with *env* from cells transfected with *env* from the HIV-1 isolates: 92/UG/092 (clade A), KNH1144 (clade A), BaL (clade B), BZ167 (clade B), NP1538 (clade B), 20635-4 (clade C), TZA246 (clade C), and D28630 (clade D). All conditions shown are after pre-incubation of the transfected HEK293T cells with 20 μg/mL of the corresponding IgG. Data reported as median fluorescence intensity (MFI) (mean, SD) from three replicates.

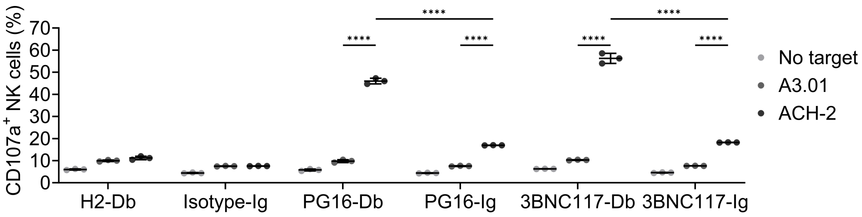

Supplementary Figure 3: PG16-Db and 3BNC117-Db elicit enhanced NK cell degranulation relative to parental IgGs. Expression of CD107a measured on $CD3^-$ $CD56^+$ NK cells either alone or co-cultured with uninfected A3.01 cells or HIV-1-infected ACH-2 cells in the presence of the H2-Db, PG16-Db, 3BNC117-Db, PG16-Ig, or 3BNC117-Ig at 2000 pM. Data reported as the percentage of $CD107a^+$ NK cells (mean, SD) from three replicates. Significance determined by two-way ANOVA followed by Dunnett's test for multiple comparisons, ****P<0.0001.

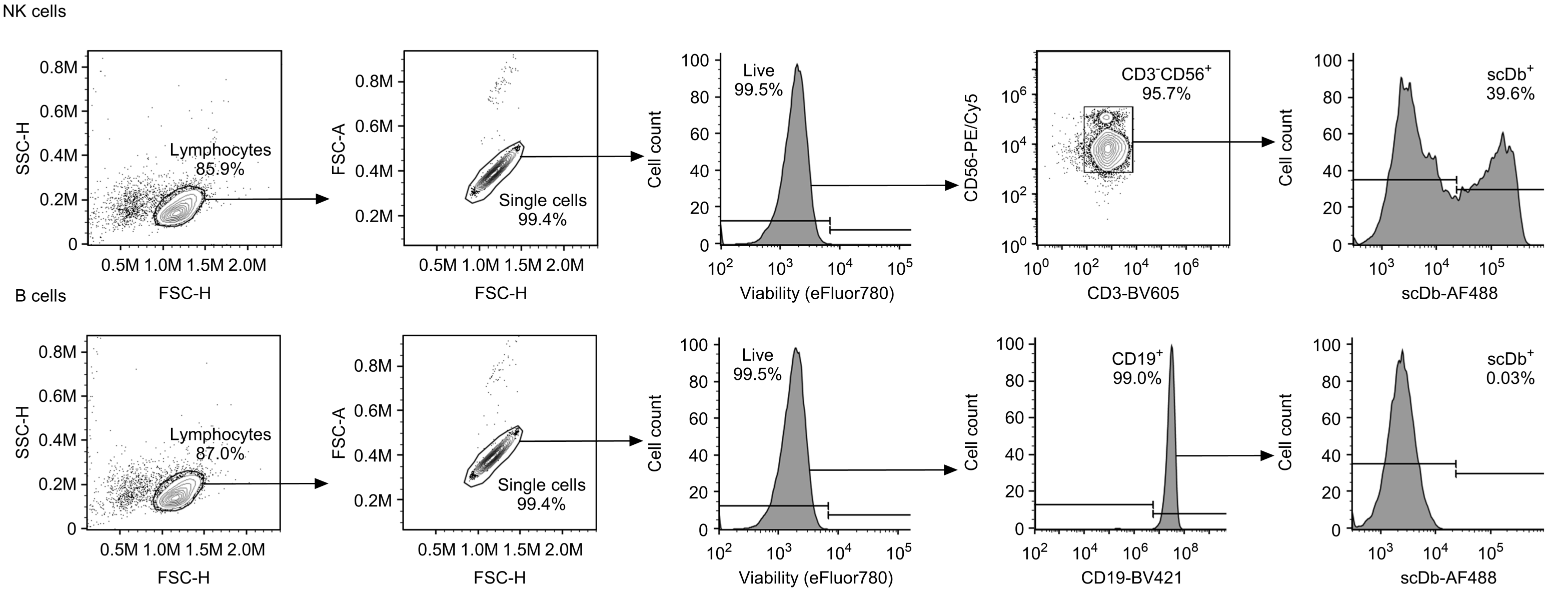

Supplementary Figure 4: Gating strategy for the NK surface CD16 binding assay. NK cells: lymphocyte size (FSC-H, SSC-H) > single cells (FSC-H, FSC-A) > live (eFlour780) > $CD3^-$ (BV605) / $CD56^+$ (PE/Cy5) > $scDb^+$ (AF488). B cells: lymphocyte size (FSC-H, SSC-H) > single cells (FSC-H, FSC-A) > live (eFlour780) > $CD19^+$ (BV421) > $scDb^+$ (AF488).

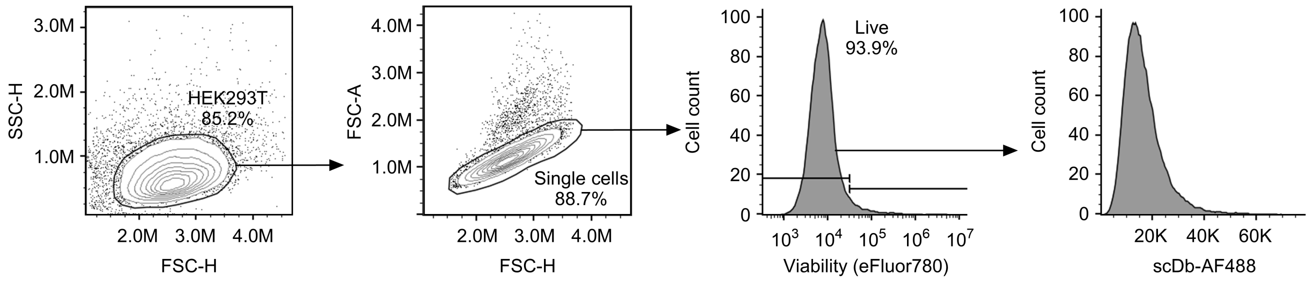

Supplementary Figure 5: Gating strategy for the surface Env binding assay. HEK293T size (FSC-H, SSC-H) > single cells (FSC-H, FSC-A) > live (eFlour780).

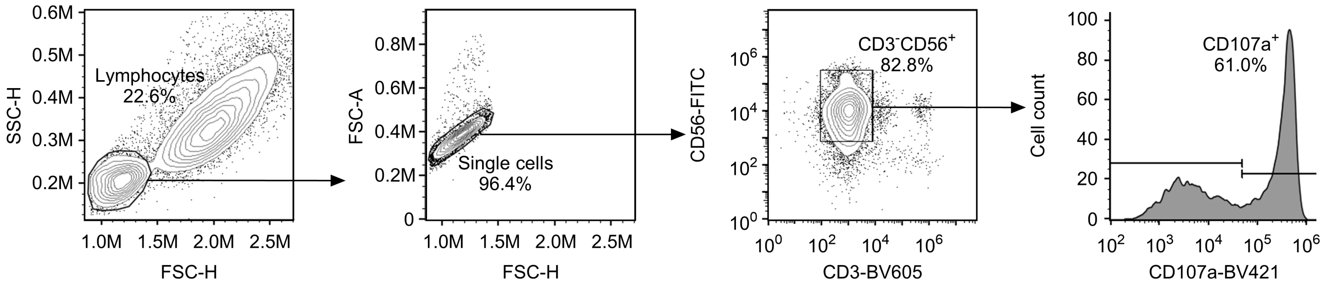

Supplementary Figure 6: Gating strategy for the NK activation co-culture. Lymphocyte size (FSC-H, SSC-H) > single cells (FSC-H, FSC-A) > live (eFlour780) > $CD3^-$ (BV605) / $CD56^+$ (FITC) > $CD107a^+$ (BV421).

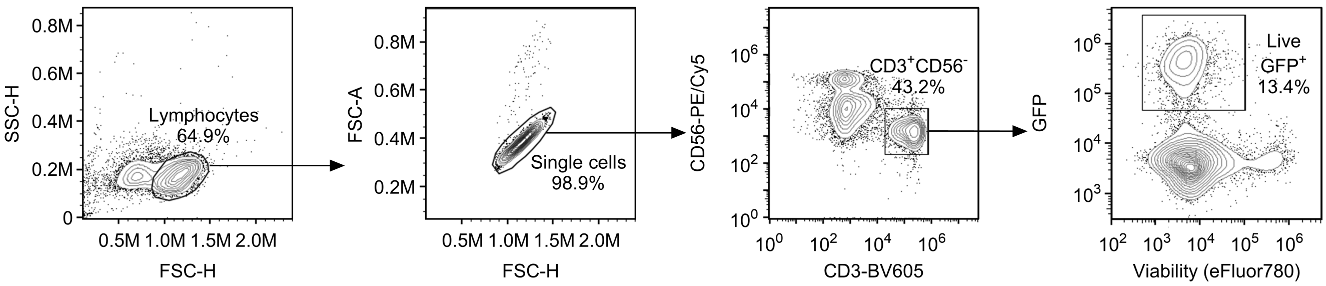

Supplementary Figure 7: Gating strategy for the *in vitro* infected cell elimination co-culture. Lymphocyte size (FSC-H, SSC-H) > single cells (FSC-H, FSC-A) > CD3+ (BV605) / CD56- (PE/Cy5) > live (eFlour780) / GFP^{+}.
