# Supplementary material for: Bispecific antibodies promote natural killer cell-mediated elimination of HIV-1 reservoir cells
Source: Nat Immunol. 2024 Jan 26;25(3):462–70. doi: 10.1038/s41590-023-01741-5 (PMC10907297; doi:10.1038/s41590-023-01741-5)
Supplement: Supplementary file 2 — Reporting Summary [file 41590_2023_1741_MOESM2_ESM.pdf]

Corresponding author(s): Janet D. Siliciano, Luis J. Montaner, Robert F. Siliciano

Last updated by author(s): Dec 21, 2023

Reporting Summary

Nature Portfolio wishes to improve the reproducibility of the work that we publish. This form provides structure for consistency and transparency in reporting. For further information on Nature Portfolio policies, see our Editorial Policies and the Editorial Policy Checklist.

Statistics

For all statistical analyses, confirm that the following items are present in the figure legend, table legend, main text, or Methods section.

n/a	Confirmed	
☐	☒	The exact sample size (*n*) for each experimental group/condition, given as a discrete number and unit of measurement
☐	☒	A statement on whether measurements were taken from distinct samples or whether the same sample was measured repeatedly
☐	☒	The statistical test(s) used AND whether they are one- or two-sided<br>*Only common tests should be described solely by name; describe more complex techniques in the Methods section.*
☒	☐	A description of all covariates tested
☐	☒	A description of any assumptions or corrections, such as tests of normality and adjustment for multiple comparisons
☐	☒	A full description of the statistical parameters including central tendency (e.g. means) or other basic estimates (e.g. regression coefficient) AND variation (e.g. standard deviation) or associated estimates of uncertainty (e.g. confidence intervals)
☐	☒	For null hypothesis testing, the test statistic (e.g. *F*, *t*, *r*) with confidence intervals, effect sizes, degrees of freedom and *P* value noted<br>*Give P values as exact values whenever suitable.*
☒	☐	For Bayesian analysis, information on the choice of priors and Markov chain Monte Carlo settings
☒	☐	For hierarchical and complex designs, identification of the appropriate level for tests and full reporting of outcomes
☐	☒	Estimates of effect sizes (e.g. Cohen's *d*, Pearson's *r*), indicating how they were calculated

Our web collection on statistics for biologists contains articles on many of the points above.

Software and code

Policy information about availability of computer code

Data collection	iQue Forecyt v9.0<br>QuantaSoft v1.7<br>QuantStudio Real-Time PCR v1.7<br>SkanIt v6.1
Data analysis	FlowJo v10.8<br>QuantaSoft Analysis Pro v1.0<br>IUPM Calculator v1.0 at https://silicianolab.johnshopkins.edu/<br>GraphPad Prism v9.5

For manuscripts utilizing custom algorithms or software that are central to the research but not yet described in published literature, software must be made available to editors and reviewers. We strongly encourage code deposition in a community repository (e.g. GitHub). See the Nature Portfolio guidelines for submitting code & software for further information.

Data

Policy information about availability of data

All manuscripts must include a data availability statement. This statement should provide the following information, where applicable:

- Accession codes, unique identifiers, or web links for publicly available datasets
- A description of any restrictions on data availability
- For clinical datasets or third party data, please ensure that the statement adheres to our policy

Data that support the findings of this study are available upon request via email to the lead corresponding author R.F.S. (rsiliciano@jhmi.edu). Data involving human research participants are subject to the data protection constraints in the written informed consent signed by the study participants.

Research involving human participants, their data, or biological material

Policy information about studies with human participants or human data. See also policy information about sex, gender (identity/presentation), and sexual orientation and race, ethnicity and racism.

Reporting on sex and gender	This study utilized blood samples from 11 HIV-1-positive male donors, and 2 female and 1 male HIV-1-negative donors. Sex was determined based on self-reporting. Sex as a biological variable was not considered in the design of this study, however, previous studies have not indicated significant sex-biased effects in regard to the HIV-1 reservoir.
Reporting on race, ethnicity, or other socially relevant groupings	Race of study participants was determined based on self-reporting. Race as a biological variable was not considered in the design of this study.
Population characteristics	n=11 HIV-1+ donors (11 males; median age=56 years, range=34-69 years; median time on ART=289 months, range=130-360 months; median time on suppressive ART=185 months, range=79-275 months; see Supplementary Table 2 for ART regimens) n=3 HIV-1- donors (2 females and 1 male; ages 29, 46, and 48)
Recruitment	All participants were enrolled on a voluntary basis and provided written informed consent.
Ethics oversight	This study was approved by the Institutional Review Boards at the University of Pennsylvania and the University of California San Francisco.

Note that full information on the approval of the study protocol must also be provided in the manuscript.

Field-specific reporting

Please select the one below that is the best fit for your research. If you are not sure, read the appropriate sections before making your selection.

☒ Life sciences ☐ Behavioural & social sciences ☐ Ecological, evolutionary & environmental sciences

For a reference copy of the document with all sections, see nature.com/documents/nr-reporting-summary-flat.pdf

Life sciences study design

All studies must disclose on these points even when the disclosure is negative.

Sample size	Sample sizes are noted for each experiment in the relevant figure legends. No statistical method was used to predetermine sample sizes. Sample sizes were determined based on availability of biological samples. Controls were included as appropriate to provide a reference to compare experimental data to.
Data exclusions	No data were excluded from any analyses.
Replication	Biological replicates (i.e. samples from different research participants or animals) were included for all ex vivo culture experiments (excluding the evaluation of different latency reversing stimuli in Figure 4, see relevant figure legends for sample sizes) and for all experiments with IL-15 Tg BLT mice (n=9 for each treatment group). Technical replicates (i.e. repeated measurements of the same sample) were included for all experiments. For all ELISA-, qPCR-, and flow cytometry-based assays, all samples were tested in triplicate. measurements All IPDA measurements were performed with 8 technical replicates. All QVOA measurements were performed with a minimum of 10 million CD4+ T cells per treatment condition. Variation between biological replicates and technical replicates for each experiment are shown where appropriate for each figure.
Randomization	For all in vitro and ex vivo culture experiments, all conditions were tested for each participant in parallel. Assignment of hIL-15TgNSG mice to treatment groups was randomized.
Blinding	During data collection and analysis for all in vitro and ex vivo culture experiments and for the hIL-15TgNSG mice experiments, the investigators were blinded to the treatment conditions of the relevant biological samples.

Reporting for specific materials, systems and methods

We require information from authors about some types of materials, experimental systems and methods used in many studies. Here, indicate whether each material, system or method listed is relevant to your study. If you are not sure if a list item applies to your research, read the appropriate section before selecting a response.

Materials & experimental systems

n/a	Involved in the study
☐	☒ Antibodies
☐	☒ Eukaryotic cell lines
☒	☐ Palaeontology and archaeology
☐	☒ Animals and other organisms
☒	☐ Clinical data
☒	☐ Dual use research of concern
☒	☐ Plants

Methods

n/a	Involved in the study
☒	☐ ChIP-seq
☐	☒ Flow cytometry
☒	☐ MRI-based neuroimaging

Antibodies

Antibodies used

NK surface CD16a binding assay
CD3 (OKT3)-BV605, Biolegend, 317321
CD19 (HIB19)-BV421, Biolegend, 302233
CD56 (MEM-188)-PE/Cy5, Biolegend, 304607
CD16 (3G8), Biolegend, 302001
6x His tag (4E3D10H2/E3)-AF488, Thermo Fisher, MA1-135-A488

Surface Env binding assay
6x His tag (4E3D10H2/E3)-AF488, Thermo Fisher, MA1-135-A488

NK activation co-culture
CD3 (OKT3)-BV605, Biolegend, 317321
CD56 (MEM-188)-FITC, Biolegend, 304603
CD107a (H4A3)-BV421, Biolegend, 328625

In vitro infected cell elimination co-culture
CD3 (OKT3)-BV421, Biolegend, 317343
CD56 (MEM-188)-PE/Cy5, Biolegend, 304607
CD3 (OKT3)-BV605, Biolegend, 317321
IgG Fc (HP6017)-BV421, 409318
p24 (ab20569)-FITC, Abcam, ab20569

Elimination of latency reversed cells from PWH co-culture
CD3 (OKT3)-BV605, Biolegend, 317321
CD56 (MEM-188)-FITC, Biolegend, 304603
CD16 (3G8)-BV421, Biolegend, 302038
NKG2D (1D11)-BV421, Biolegend, 320822
Siglec-7 (6-434)-PerCP/Cy5.5, Biolegend, 339216
CD57 (HNK-1)-PerCP/Cy5.5, Biolegend, 359622
PD-1 (EH12.2H7)-PE/Cy5, Biolegend, 329971

Generation of the hIL-15TG NSG mice
mCD45 (30-F11)-AF700, BD Biosciences, 560510
hCD45 (HI30)-FITC, BD Biosciences, 555482
hCD3 (UCHT1)-BUV805, BD Biosciences, 612895
hCD4 (SK3)-BUV395, BD Biosciences, 563550
hCD8 (SK1)-PerCP-Cy5.5, BD Biosciences, 565310
hCD56 (NCAM16.2)-BV650, BD Biosciences, 564057

Measuring markers of T cell and NK cell activation and maturation
mCD45 (30-F11)-AF700, BD Biosciences, 560510
hCD45 (HI30)-FITC, BD Biosciences, 555482
hCD3 (UCHT1)-BUV805, BD Biosciences, 612895
hCD4 (SK3)-BUV395, BD Biosciences, 563550
hCD8 (SK1)-PerCP-Cy5.5, BD Biosciences, 565310
hCD56 (NCAM16.2)-BV650, BD Biosciences, 564057
HLA-DR (L243)-APC, BD Biosciences, 340691
hCD38 (HIT2)-PE, BD Biosciences, 555460
hCD57 (NK-1)-PE-CF594, BD Biosciences, 562488

	Unless specified otherwise in the Methods, all antibodies were used at a 1:50 dilution for staining.
Validation	All antibodies used have a validated technical technical data sheet as per the manufacturers' websites.

Eukaryotic cell lines

Policy information about cell lines and Sex and Gender in Research

Cell line source(s)	HEK293T cells were purchased from ATCC (catalog number CRL-3216). A3.01 and ACH-2 cells were obtained from the NIH HIV Reagents Program (catalog numbers ARP-166 and ARP-349 respectively).
Authentication	We confirmed with commercial and collaborative sources from where the cells were obtained that cell lines were authentic and free of contamination.
Mycoplasma contamination	All cell line tested negative for mycoplasma contamination prior to use.
Commonly misidentified lines (See ICLAC register)	No commonly misidentified cell lines were used.

Animals and other research organisms

Policy information about studies involving animals; ARRIVE guidelines recommended for reporting animal research, and Sex and Gender in Research

Laboratory animals	NSG-Tg(hIL-15) (NOD.Cg-Prkdcscid Il2rgtm1Wjl Tg(IL15)1Sz/SzJ, Jackson Laboratory) mice, 6-8 weeks old at time of surgery. All animals recruited in this study were housed in the Wistar Institute humanized mice holding room with a 12 hour light/dark cycle at temperatures of 20-23°C and 40-60% humidity.
Wild animals	No wild animals were used.
Reporting on sex	All mice used in this study were female.
Field-collected samples	No field-collected samples were used.
Ethics oversight	The Wistar Institute Animal Care and Research Committee (protocol# 201360).

Note that full information on the approval of the study protocol must also be provided in the manuscript.

Flow Cytometry

Plots

Confirm that:

☒ The axis labels state the marker and fluorochrome used (e.g. CD4-FITC).

☒ The axis scales are clearly visible. Include numbers along axes only for bottom left plot of group (a 'group' is an analysis of identical markers).

☒ All plots are contour plots with outliers or pseudocolor plots.

☒ A numerical value for number of cells or percentage (with statistics) is provided.

Methodology

Sample preparation	The NK, B, and CD4+ T cells used for flow cytometry were isolated from healthy participant PBMCs by immunomagnetic negative selection (Stemcell).
Instrument	Intellicyt iQue Screener Plus (Sartorius), Violet/Blue/Red lasers
Software	FlowJo v10.8 (BD Bioscience) was used for data analysis.
Cell population abundance	The median intensities or positive fractions were shown in each relevant figure.
Gating strategy	NK surface CD16a binding assay<br>NK cells: lymphocyte size (FSC-H, SSC-H) > single cells (FSC-H, FSC-A) > live (eFlour780) > CD3- (BV605) / CD56+ (PE/Cy5) > scDb+ (AF488)<br>B cells: lymphocyte size (FSC-H, SSC-H) > single cells (FSC-H, FSC-A) > live (eFlour780) > CD19+ (BV421) > scDb+ (AF488)<br><br>Surface Env binding assay<br>HEK293T size (FSC-H, SSC-H) > single cells (FSC-H, FSC-A) > live (eFlour780)<br><br>NK activation co-culture

lymphocyte size (FSC-H, SSC-H) > single cells (FSC-H, FSC-A) > live (eFlour780) > CD3- (BV605) / CD56+ (FITC) > CD107a+ (BV421)

In vitro infected cell elimination co-culture
lymphocyte size (FSC-H, SSC-H) > single cells (FSC-H, FSC-A) > CD3+ (BV605) / CD56- (PE/Cy5) > live (eFlour780) / GFP+

☒ Tick this box to confirm that a figure exemplifying the gating strategy is provided in the Supplementary Information.
